# Supplementary material for: Whole exome sequencing analysis identifies genes for alcohol consumption
Source: Nat Commun. 2024 Jul 10;15:5777. doi: 10.1038/s41467-024-50132-3 (PMC11233704; doi:10.1038/s41467-024-50132-3)
Supplement: Supplementary file 1 — Supplementary Information [file 41467_2024_50132_MOESM1_ESM.pdf]

Supplementary Information For

Whole exome sequencing analysis identifies genes for alcohol consumption

Jujiao Kang[1,2,†], Yue-Ting Deng[3,†], Bang-Sheng Wu[3,†], Wei-Shi Liu[3], Ze-Yu Li[1,2], Shitong Xiang[1,2], Liu Yang[3], Jia You[1,2], Xiaohong Gong[4], Tianye Jia[1,2], Jin-Tai Yu[3,*], Wei Cheng[1,2,3,5,*], Jianfeng Feng[1,2,5,6,*]

[1] Institute of Science and Technology for Brain-Inspired Intelligence (ISTBI), Fudan University, Shanghai 200433, P.R. China.

[2] Key Laboratory of Computational Neuroscience and Brain-Inspired Intelligence (Fudan University), Ministry of Education, Shanghai 200433, P.R. China.

[3] Department of Neurology and National Center for Neurological Disorders, Huashan Hospital, State Key Laboratory of Medical Neurobiology and MOE Frontiers Center for Brain Science, Shanghai Medical College, Fudan University, Shanghai 200433, P.R. China.

[4] School of Life Sciences, Fudan University, Shanghai 200433, P.R. China.

[5] Fudan ISTBI—ZJNU Algorithm Centre for Brain-inspired Intelligence, Zhejiang Normal University, Zhejiang, China

[6] Department of Computer Science, University of Warwick, Coventry CV4 7AL, UK

†These authors contributed equally to this work.

* Addresses correspondence to Prof. Jian-Feng Feng, Institute of Science and Technology for Brain-Inspired Intelligence, Fudan University, Shanghai 200433, China; Prof. Wei Cheng, Institute of Science and Technology for Brain-Inspired Intelligence, Fudan University, Shanghai 200433, China; Prof. Jin-Tai Yu, Department of Neurology and Institute of Neurology, Huashan Hospital, Shanghai Medical College, Fudan University, 12th Wulumuqi Zhong Road, Shanghai 200040, China

E-mail addresses: jffeng@fudan.edu.cn (JF Feng); wcheng@fudan.edu.cn (W Cheng); jintai_yu@fudan.edu.cn (JT Yu).

Tel: +86 21 52888160; Fax: +86 21 62483421.

Content

Supplementary Fig. 1 Quantile-Quantile plot of single-variant association analysis of alcohol consumption. 7

Supplementary Fig. 2 Manhattan and Quantile-Quantile plot of single-variant association analysis of AUDIT-T. 8

Supplementary Fig. 3 Manhattan and Quantile-Quantile plot of single-variant association analysis of AUDIT-C. 9

Supplementary Fig. 4 Manhattan and Quantile-Quantile plot of single-variant association analysis of AUDIT-P. 10

Supplementary Fig. 5 Manhattan and Quantile-Quantile plot of gene-based collapsing association analysis of alcohol consumption. 11

Supplementary Fig. 6 Manhattan and Quantile-Quantile plot of gene-based collapsing association analysis of AUDIT-T. 12

Supplementary Fig. 7 Manhattan and Quantile-Quantile plot of gene-based collapsing association analysis of AUDIT-C. 13

Supplementary Fig. 8 Manhattan and Quantile-Quantile plot of gene-based collapsing association analysis of AUDIT-P. 14

Supplementary Fig. 9 Leave-one-variant-out (LOVO) analysis of the *ADH1C* gene. 15

Supplementary Fig. 10 Leave-one-variant-out (LOVO) analysis of the *ADH1A* gene. 16

Supplementary Fig. 11 Leave-one-variant-out (LOVO) analysis of the *ADH5* gene..17

Supplementary Fig. 12 Leave-one-variant-out (LOVO) analysis of the *GIGYF1* gene. 18
Supplementary Fig. 13 Leave-one-variant-out (LOVO) analysis of the *HECTD4* gene. 20
Supplementary Fig. 14 Leave-one-variant-out (LOVO) analysis of the *ANKRD12* gene. 21
Supplementary Fig. 15 Leave-one-variant-out (LOVO) analysis of the *APC2* gene. .23
Supplementary Fig. 16 Leave-one-variant-out (LOVO) analysis of the *KDM5B* gene. 24
Supplementary Fig. 17 Leave-one-variant-out (LOVO) analysis of the *CTNNA2* gene. 25
Supplementary Fig. 18 Leave-one-variant-out (LOVO) analysis of the *SNX17* gene. 26
Supplementary Fig. 19 Leave-one-variant-out (LOVO) analysis of the *LGI2* gene. ..27
Supplementary Fig. 20 Leave-one-variant-out (LOVO) analysis of the *ATP1A2* gene. 28
Supplementary Fig. 21 Tissue-specificity analysis of the alcohol consumption genes. 29
Supplementary Fig. 22 Expression of genes specifically enriched in liver. 30
Supplementary Fig. 23 Expression of the *GIGYF1* and *ANKRD12* gene. 31
Supplementary Fig. 24 Cell-type expression of the *ANKRD12* and *GIGYF1* gene in the brain. 32

Supplementary Fig. 25 PheWAS results of *ANKRD12*.......33

Supplementary Fig. 26 PheWAS results of *GIGYF1*.......35

Supplementary Fig. 27 PheWAS results of *ADH1C*.......36

Supplementary Fig. 28 PheWAS results of *ADH1A*.......37

Supplementary Fig. 29 PheWAS results of *ADH5*.......38

Supplementary Fig. 30 PheWAS results of *HECTD4*.......39

Supplementary Fig. 31 PheWAS results of *APC2*.......40

Supplementary Fig. 32 PheWAS results of *SNX17*.......41

Supplementary Fig. 33 PheWAS results of *LGI2*.......42

Supplementary Fig. 34 PheWAS results of *CTNNA2*.......43

Supplementary Fig. 35 PheWAS results of *KDM5B*.......44

Supplementary Fig. 36 PheWAS results of *ATP1A2*.......45

Supplementary Fig. 37 PheWAS results of variant chr2:27130762:G:C.......46

Supplementary Fig. 38 PheWAS results of variant chr2:27433955:G:GA.......47

Supplementary Fig. 39 PheWAS results of variant chr2:27579938:A:G.......48

Supplementary Fig. 40 PheWAS results of variant chr4:3228683:AGAG:A.......49

Supplementary Fig. 41 PheWAS results of variant chr4:39275003:A:AG.......50

Supplementary Fig. 42 PheWAS results of variant chr4:39437644:T:C.......51

Supplementary Fig. 43 PheWAS results of variant chr4:39448609:C:A.......52

Supplementary Fig. 44 PheWAS results of variant chr4:39527132:G:A.......53

Supplementary Fig. 45 PheWAS results of variant chr4:99284715:C:G.......54

Supplementary Fig. 46 PheWAS results of variant chr4:99347033:C:A....................55
Supplementary Fig. 47 PheWAS results of variant chr4:99347888:G:A. 56
Supplementary Fig. 48 PheWAS results of variant chr4:99611445:G:A....................57
Supplementary Fig. 49 PheWAS results of variant chr4:102267552:C:T. 58
Supplementary Fig. 50 PheWAS results of variant chr7:73599571:C:T. 60
Supplementary Fig. 51 PheWAS results of variant chr11:47410415:C:T...................61
Supplementary Fig. 52 PheWAS results of variant chr11:113368360:G:A................62
Supplementary Fig. 53 PheWAS results of variant chr11:113400106:G:A................63
Supplementary Fig. 54 PheWAS results of variant chr12:81253692:T:A. 64
Supplementary Fig. 55 PheWAS results of variant chr14:94378610:C:T. 65
Supplementary Fig. 56 PheWAS results of variant chr15:76339947:G:T. 67
Supplementary Fig. 57 PheWAS results of variant chr16:28825882:T:G. 68
Supplementary Fig. 58 PheWAS results of variant chr16:30082458:C:G..................69
Supplementary Fig. 59 PheWAS results of variant chr17:45816385:A:G. 70
Supplementary Fig. 60 PheWAS results of variant chr18:23529286:T:C. 73
Supplementary Fig. 61 PheWAS results of variant chr19:48702915:C:T. 74

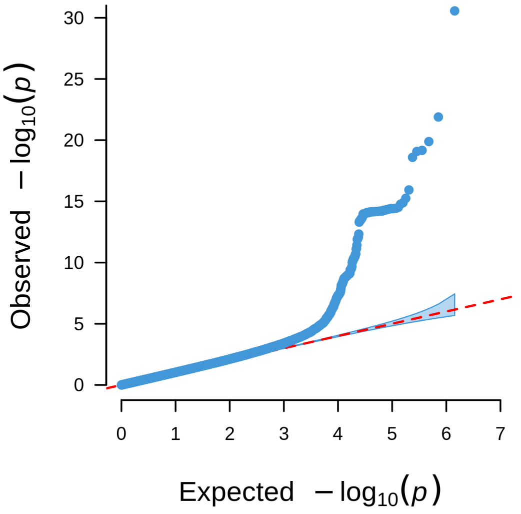

Supplementary Fig. 1 Quantile-Quantile plot of single-variant association analysis of alcohol consumption.

The *x* axis represented the expected -$\log_{10}(P)$. and the *y* axis represented the observed -$\log_{10}(P)$ of the association between single variant and alcohol consumption (N = 304,119 biologically independent samples).

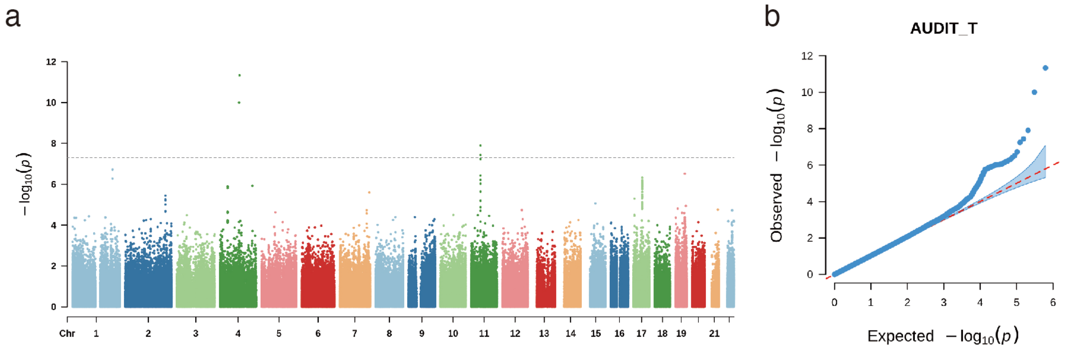

Supplementary Fig. 2 Manhattan and Quantile-Quantile plot of single-variant association analysis of AUDIT-T.

a. The *x* axis represented the chromosome and the *y* axis represented the -$\log_{10}(P)$ of the association between single variant and AUDIT total score (N = 101,240 biologically independent samples). P-values shown are two-sided and unadjusted for multiple testing. b. The *x* axis represented the expected -$\log_{10}(P)$. and the *y* axis represented the observed -$\log_{10}(P)$.

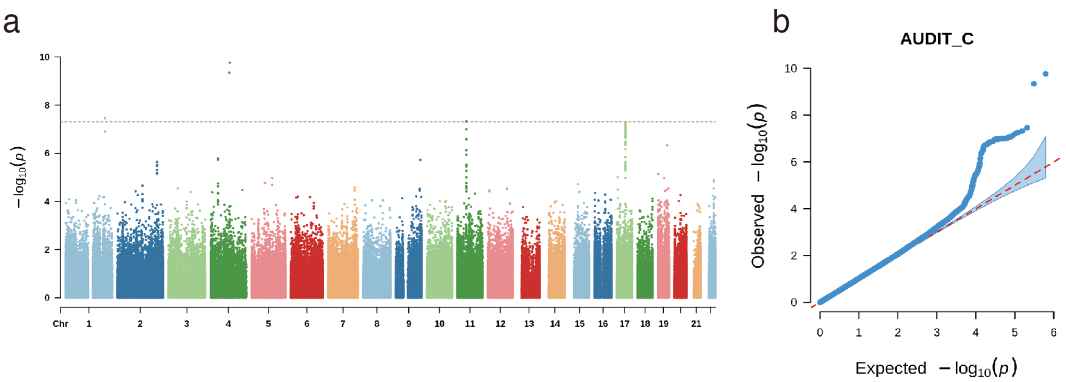

Supplementary Fig. 3 Manhattan and Quantile-Quantile plot of single-variant association analysis of AUDIT-C.

a. The *x* axis represented the chromosome and the *y* axis represented the -$\log_{10}(P)$ of the association between single variant and AUDIT consumption score (N = 101,240 biologically independent samples). P-values shown are two-sided and unadjusted for multiple testing. b. The *x* axis represented the expected -$\log_{10}(P)$. and the *y* axis represented the observed -$\log_{10}(P)$.

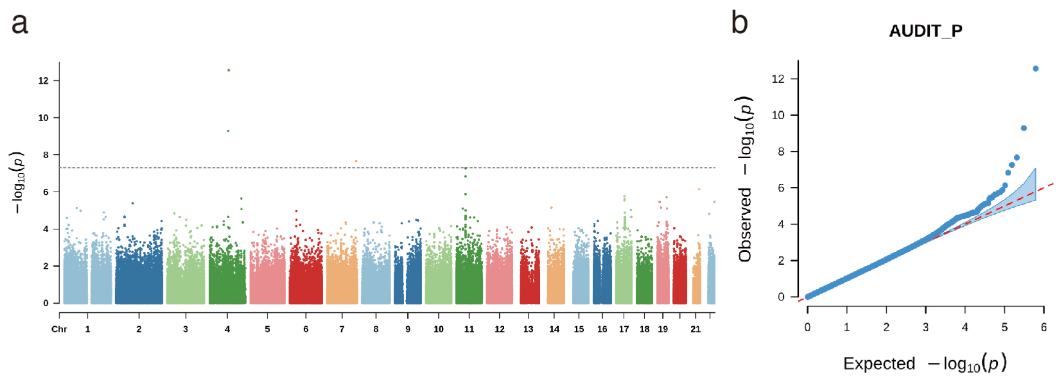

Supplementary Fig. 4 Manhattan and Quantile-Quantile plot of single-variant association analysis of AUDIT-P.

a. The x axis represented the chromosome and the y axis represented the $-\log_{10}(P)$ of the association between single variant and AUDIT problematic use score (N = 101,240 biologically independent samples). P-values shown are two-sided and unadjusted for multiple testing. b. The x axis represented the expected $-\log_{10}(P)$. and the y axis represented the observed $-\log_{10}(P)$.

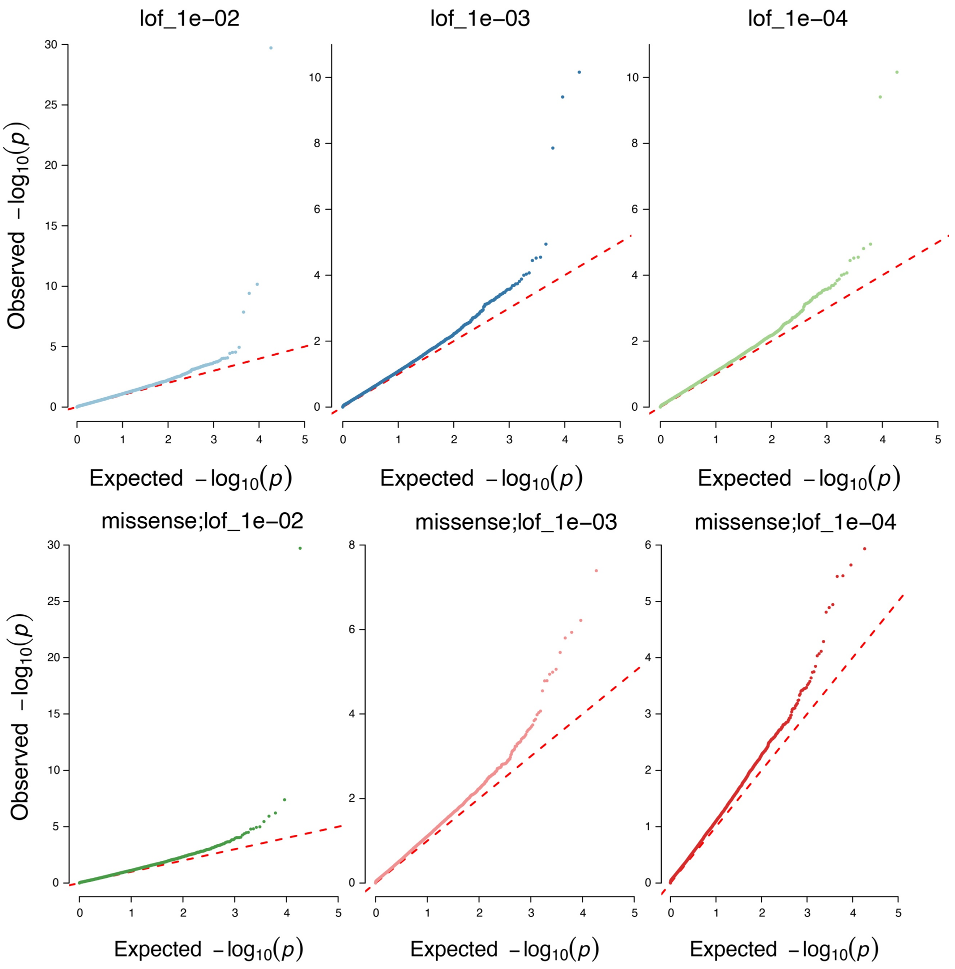

Supplementary Fig. 5 Manhattan and Quantile-Quantile plot of gene-based collapsing association analysis of alcohol consumption.

The *x* axis represented the expected -$\log_{10}(P)$. and the *y* axis represented the observed -$\log_{10}(P)$ of the gene-based collapsing analysis of the alcohol consumption (N = 304,119 biologically independent samples). Combinations of different MAF groups and consequence groups (lof and missense) were used.

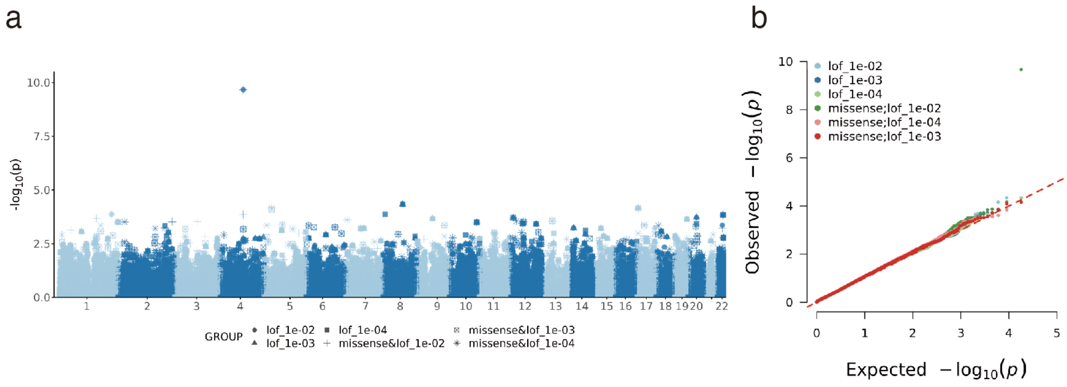

Supplementary Fig. 6 Manhattan and Quantile-Quantile plot of gene-based collapsing association analysis of AUDIT-T.

a. The x axis represented the chromosome and the y axis represented the -$\log_{10}(P)$ of the gene-based collapsing analysis of the AUDIT total score (N = 101,240 biologically independent samples). P-values shown are two-sided and unadjusted for multiple testing. The shape indicates the combination of different MAF groups and consequence groups, including the lof and missense. b. The x axis represented the expected -$\log_{10}(P)$. and the y axis represented the observed -$\log_{10}(P)$. The color indicates the combination of different MAF groups and consequence groups, including the lof and missense.

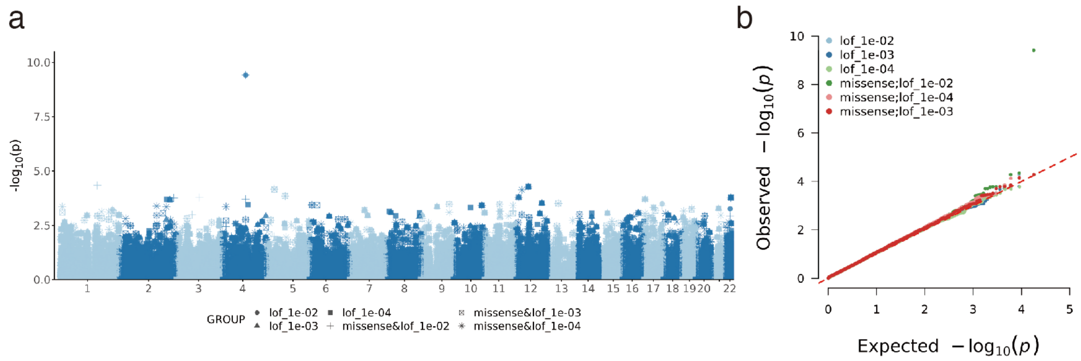

Supplementary Fig. 7 Manhattan and Quantile-Quantile plot of gene-based collapsing association analysis of AUDIT-C.

a. The *x* axis represented the chromosome and the *y* axis represented the -$\log_{10}(P)$ of the gene-based collapsing analysis of the AUDIT consumption score (N = 101,240 biologically independent samples). P-values shown are two-sided and unadjusted for multiple testing. The shape indicates the combination of different MAF groups and consequence groups, including the lof and missense. b. The *x* axis represented the expected -$\log_{10}(P)$. and the *y* axis represented the observed -$\log_{10}(P)$. The color indicates the combination of different MAF groups and consequence groups, including the lof and missense.

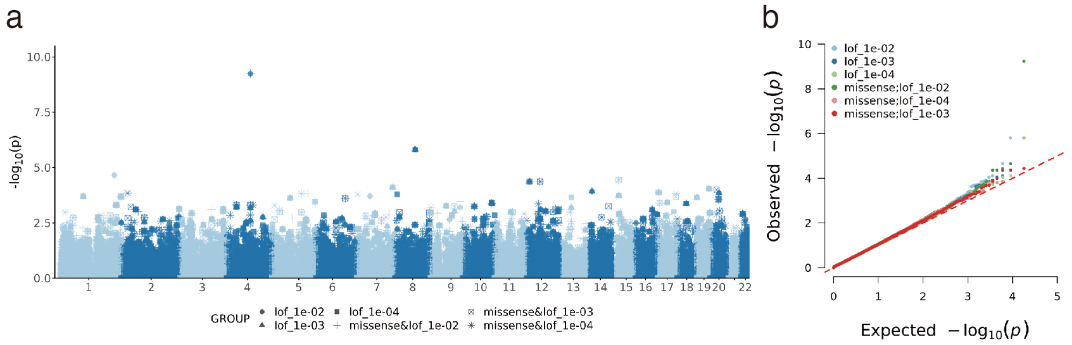

Supplementary Fig. 8 Manhattan and Quantile-Quantile plot of gene-based collapsing association analysis of AUDIT-P.

a. The *x* axis represented the chromosome and the *y* axis represented the -$\log_{10}(P)$ of the gene-based collapsing analysis of the AUDIT problematic use score (N = 101,240 biologically independent samples). P-values shown are two-sided and unadjusted for multiple testing. The shape indicates the combination of different MAF groups and consequence groups, including the lof and missense. b. The *x* axis represented the expected -$\log_{10}(P)$. and the *y* axis represented the observed -$\log_{10}(P)$. The color indicates the combination of different MAF groups and consequence groups, including the lof and missense.

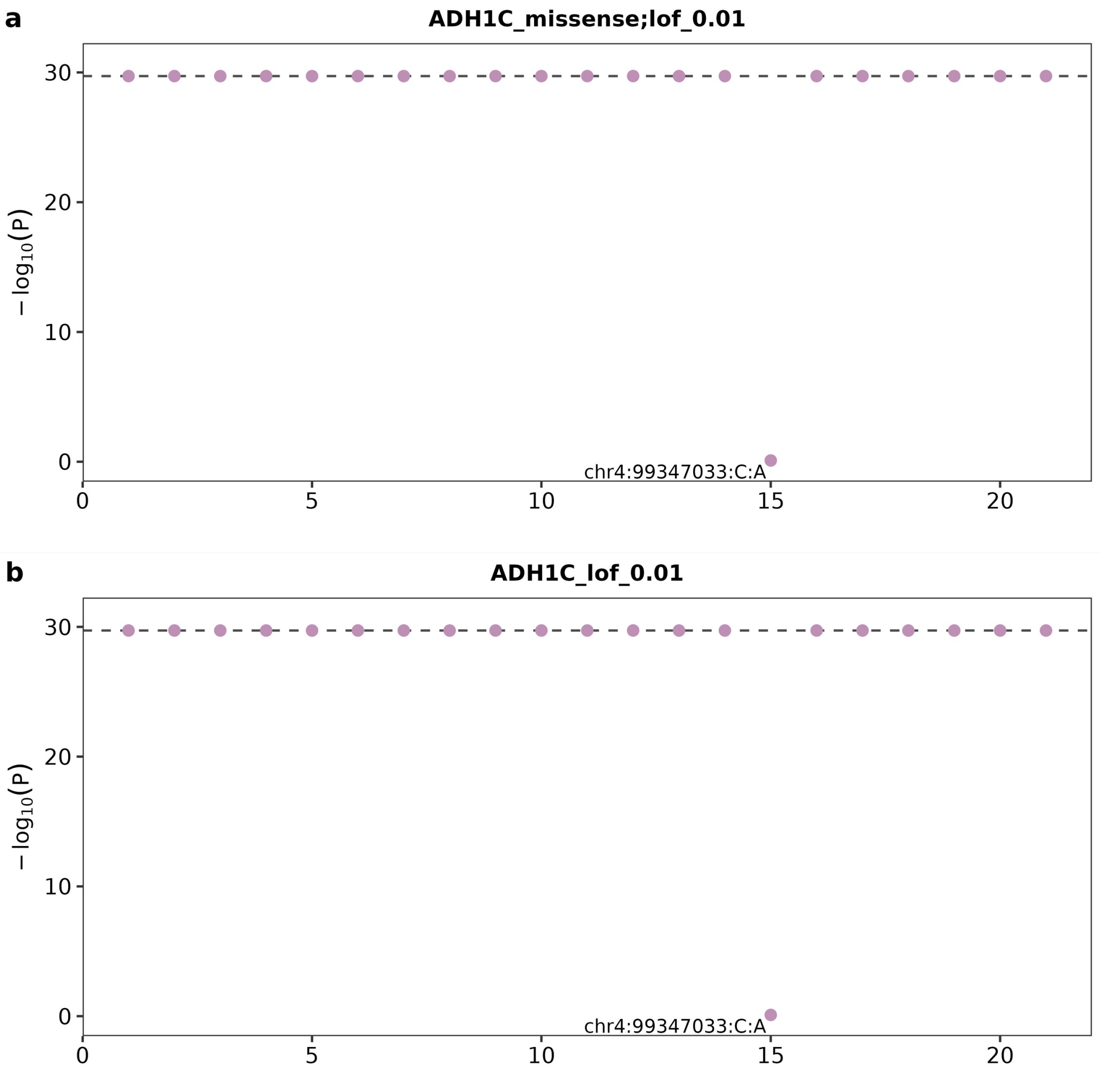

Supplementary Fig. 9 Leave-one-variant-out (LOVO) analysis of the *ADH1C* gene.

a. Results of the LOVO analysis of the *ADH1C* gene in the LOF plus missense and max MAF<0.01 group. The *x* axis represents a single variant removed from the gene-based analysis, while the *y* axis represented the -$\log_{10}(P)$ of the association without that given variant. P-values shown are two-sided and unadjusted for multiple testing. b. Results of the LOVO analysis of the *ADH1C* gene in the LOF and max MAF<0.01 group.

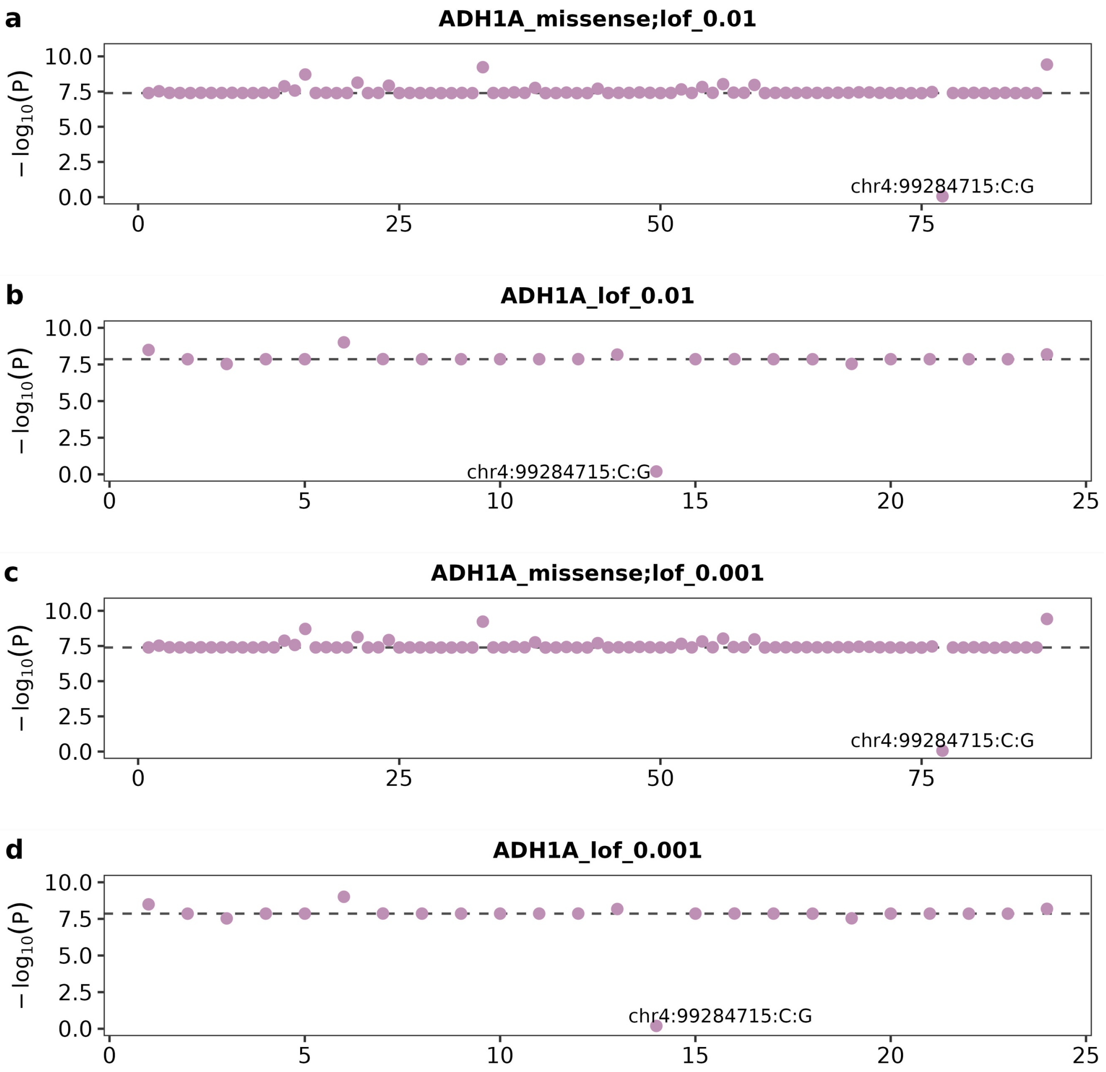

Supplementary Fig. 10 Leave-one-variant-out (LOVO) analysis of the *ADH1A* gene.

a. Results of the LOVO analysis of the *ADH1A* gene in the LOF plus missense and max MAF<0.01 group. The *x* axis represents a single variant removed from the gene-based analysis, while the *y* axis represented the -$\log_{10}(P)$ of the association without that given variant. P-values shown are two-sided and unadjusted for multiple testing. b. Results of the LOVO analysis of the *ADH1A* gene in the LOF and max MAF<0.01 group. c. Results of the LOVO analysis of the *ADH1A* gene in the LOF plus missense and max MAF<0.001 group. d. Results of the LOVO analysis of the *ADH1A* gene in the LOF and max MAF<0.001 group.

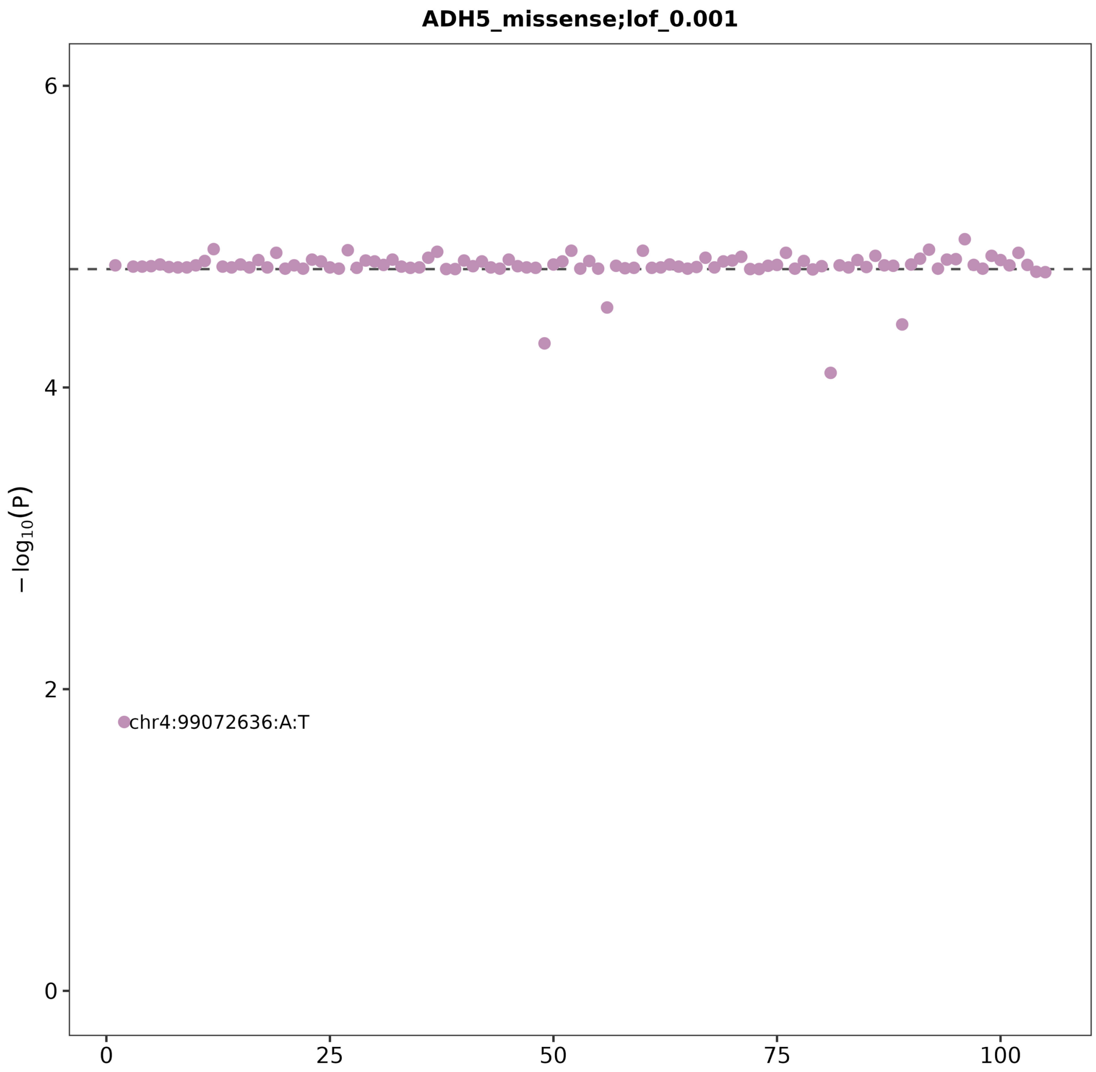

Supplementary Fig. 11 Leave-one-variant-out (LOVO) analysis of the *ADH5* gene.

Results of the LOVO analysis of the *ADH5* gene in the LOF plus missense and max MAF<0.001 group. The *x* axis represents a single variant removed from the gene-based analysis, while the *y* axis represented the -$\log_{10}(P)$ of the association without that given variant. P-values shown are two-sided and unadjusted for multiple testing.

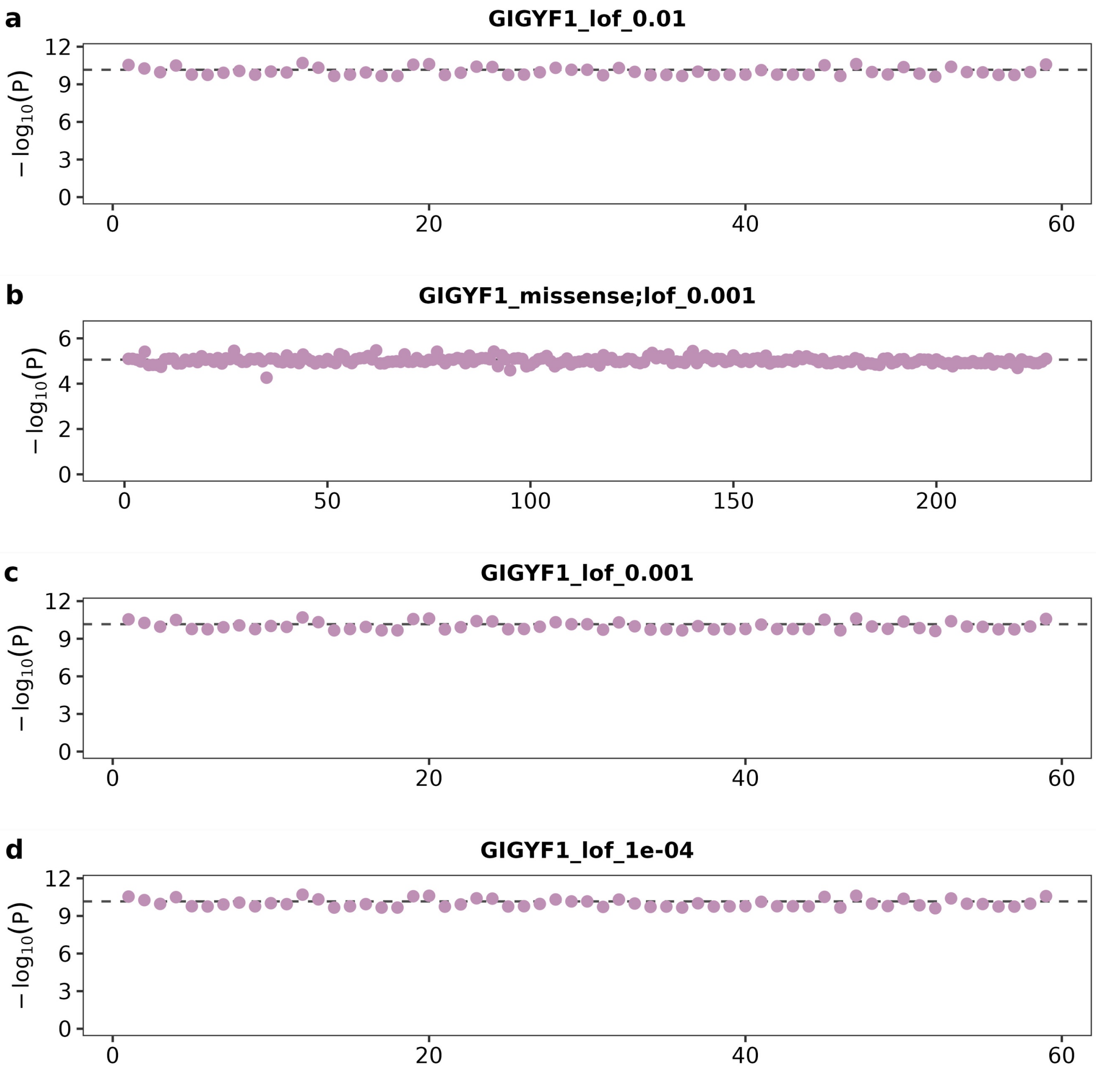

Supplementary Fig. 12 Leave-one-variant-out (LOVO) analysis of the *GIGYF1* gene.

a. Results of the LOVO analysis of the *GIGYF1* gene in the LOF and max MAF<0.01 group. The *x* axis represents a single variant removed from the gene-based analysis, while the *y* axis represented the -$\log_{10}(P)$ of the association without that given variant. P-values shown are two-sided and unadjusted for multiple testing. b. Results of the LOVO analysis of the *GIGYF1* gene in the LOF plus missense and max MAF<0.001 group. c. Results of the LOVO analysis of the *GIGYF1* gene in the LOF and max MAF<0.001 group. d. Results of the LOVO analysis of the *GIGYF1* gene in the LOF and max

MAF<0.0001 group.

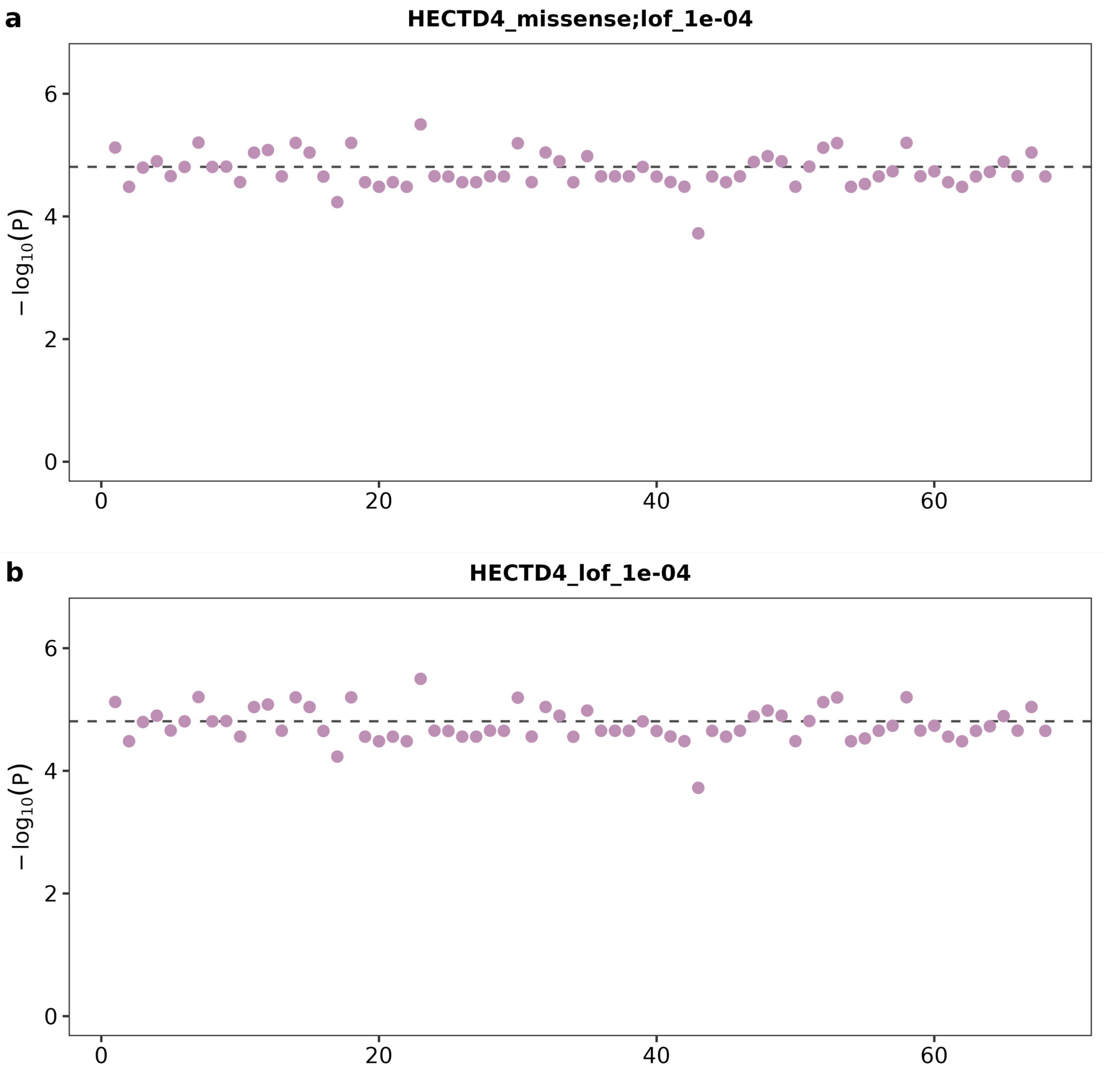

Supplementary Fig. 13 Leave-one-variant-out (LOVO) analysis of the *HECTD4* gene.

a. Results of the LOVO analysis of the *HECTD4* gene in the LOF plus missense and max MAF<0.0001 group. The *x* axis represents a single variant removed from the gene-based analysis, while the *y* axis represented the -$\log_{10}(P)$ of the association without that given variant. P-values shown are two-sided and unadjusted for multiple testing. b. Results of the LOVO analysis of the *HECTD4* gene in the LOF and max MAF<0.0001 group.

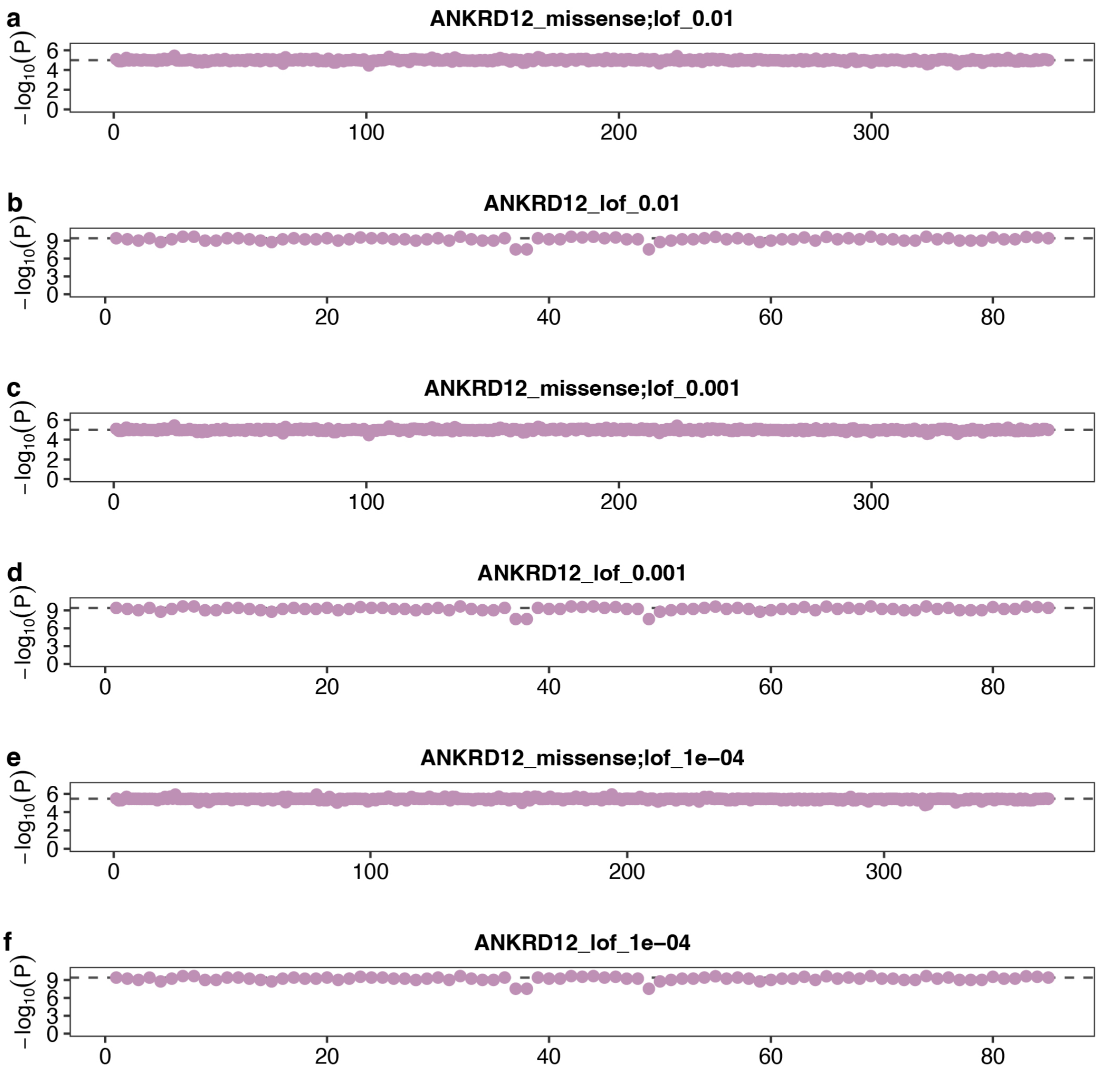

Supplementary Fig. 14 Leave-one-variant-out (LOVO) analysis of the *ANKRD12* gene.

a. Results of the LOVO analysis of the *ANKRD12* gene in the LOF plus missense and max MAF<0.01 group. The *x* axis represents a single variant removed from the gene-based analysis, while the *y* axis represented the $-\log_{10}(P)$ of the association without that given variant. P-values shown are two-sided and unadjusted for multiple testing. b. Results of the LOVO analysis of the *ANKRD12* gene in the LOF and max MAF<0.01 group. c. Results of the LOVO analysis of the *ANKRD12* gene in the LOF plus missense and max MAF<0.001 group. d. Results of the LOVO analysis of the *ANKRD12* gene in the LOF and max MAF<0.001 group. e. Results of

the LOVO analysis of the *ANKRD12* gene in the LOF plus missense and max MAF<0.0001 group. f. Results of the LOVO analysis of the *ANKRD12* gene in the LOF and max MAF<0.0001 group.

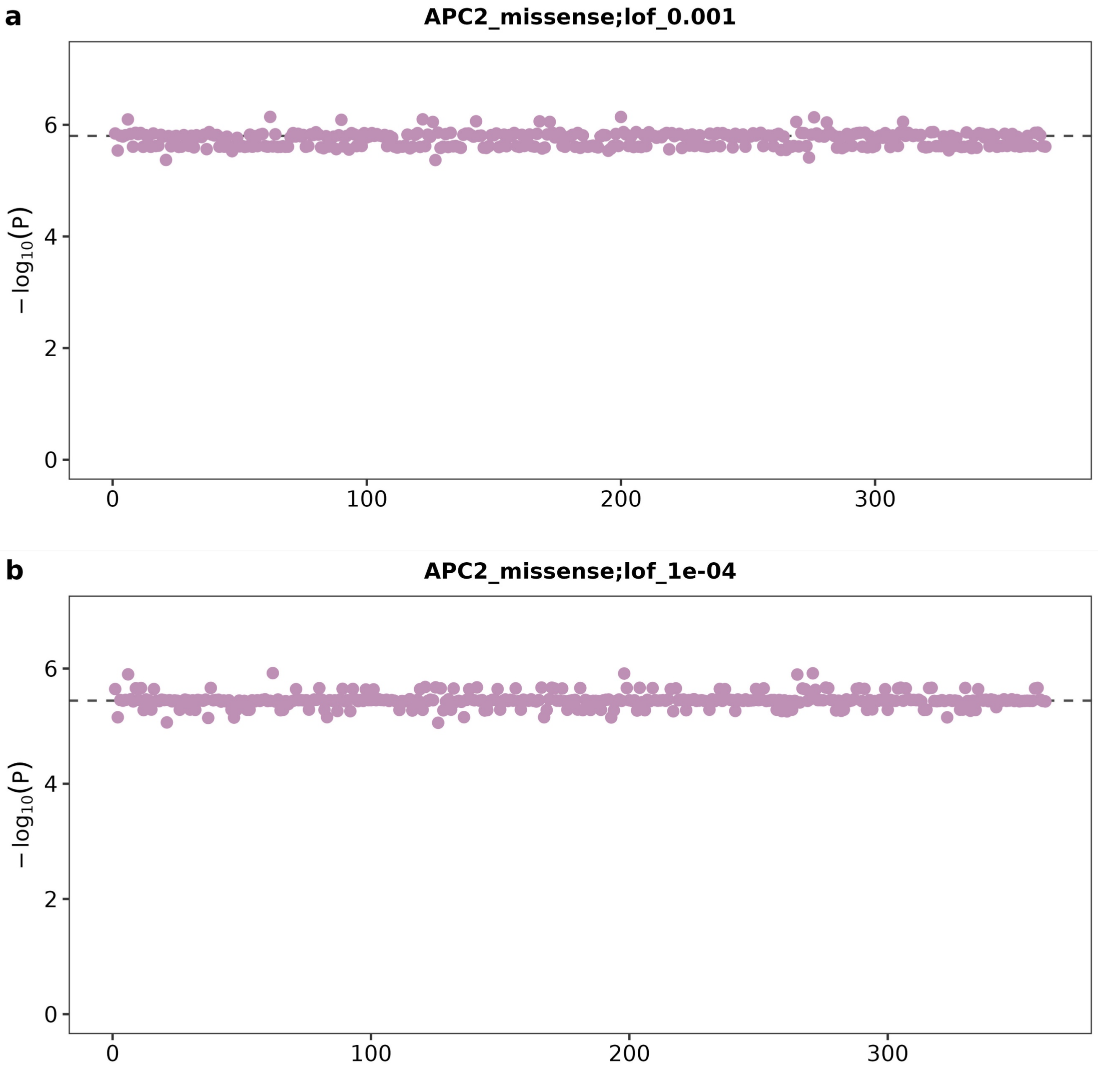

Supplementary Fig. 15 Leave-one-variant-out (LOVO) analysis of the *APC2* gene.

a. Results of the LOVO analysis of the *APC2* gene in the LOF plus missense and max MAF<0.001 group. The *x* axis represents a single variant removed from the gene-based analysis, while the *y* axis represented the $-\log_{10}(P)$ of the association without that given variant. P-values shown are two-sided and unadjusted for multiple testing. b. Results of the LOVO analysis of the *APC2* gene in the LOF plus missense and max MAF<0.0001 group.

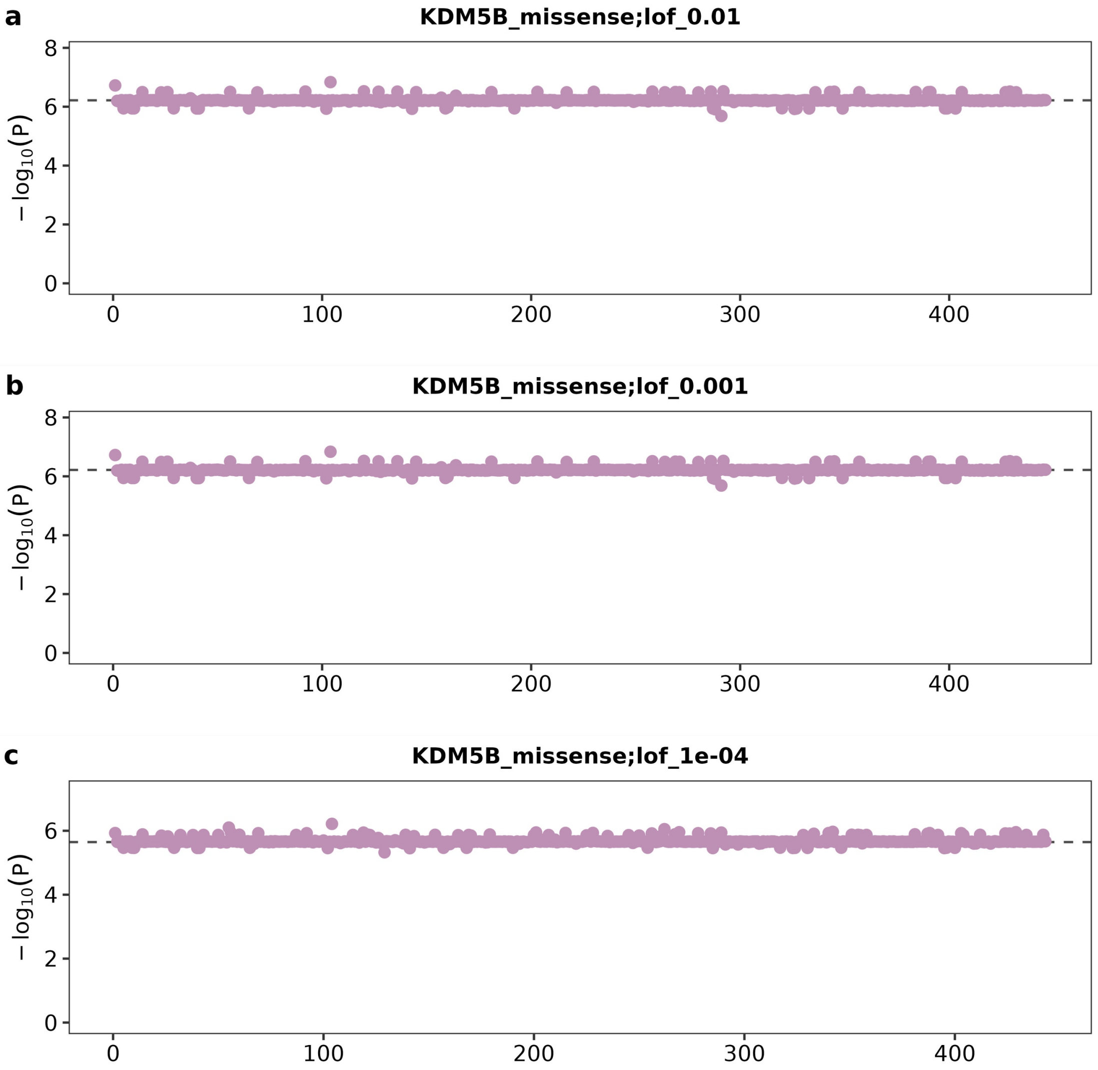

Supplementary Fig. 16 Leave-one-variant-out (LOVO) analysis of the *KDM5B* gene.

a. Results of the LOVO analysis of the *KDM5B* gene in the LOF plus missense and max MAF<0.01 group. The x axis represents a single variant removed from the gene-based analysis, while the y axis represented the $-\log_{10}(P)$ of the association without that given variant. P-values shown are two-sided and unadjusted for multiple testing. b. Results of the LOVO analysis of the *KDM5B* gene in the LOF plus missense and max MAF<0.001 group. c. Results of the LOVO analysis of the *KDM5B* gene in the LOF plus missense and max MAF<0.0001 group.

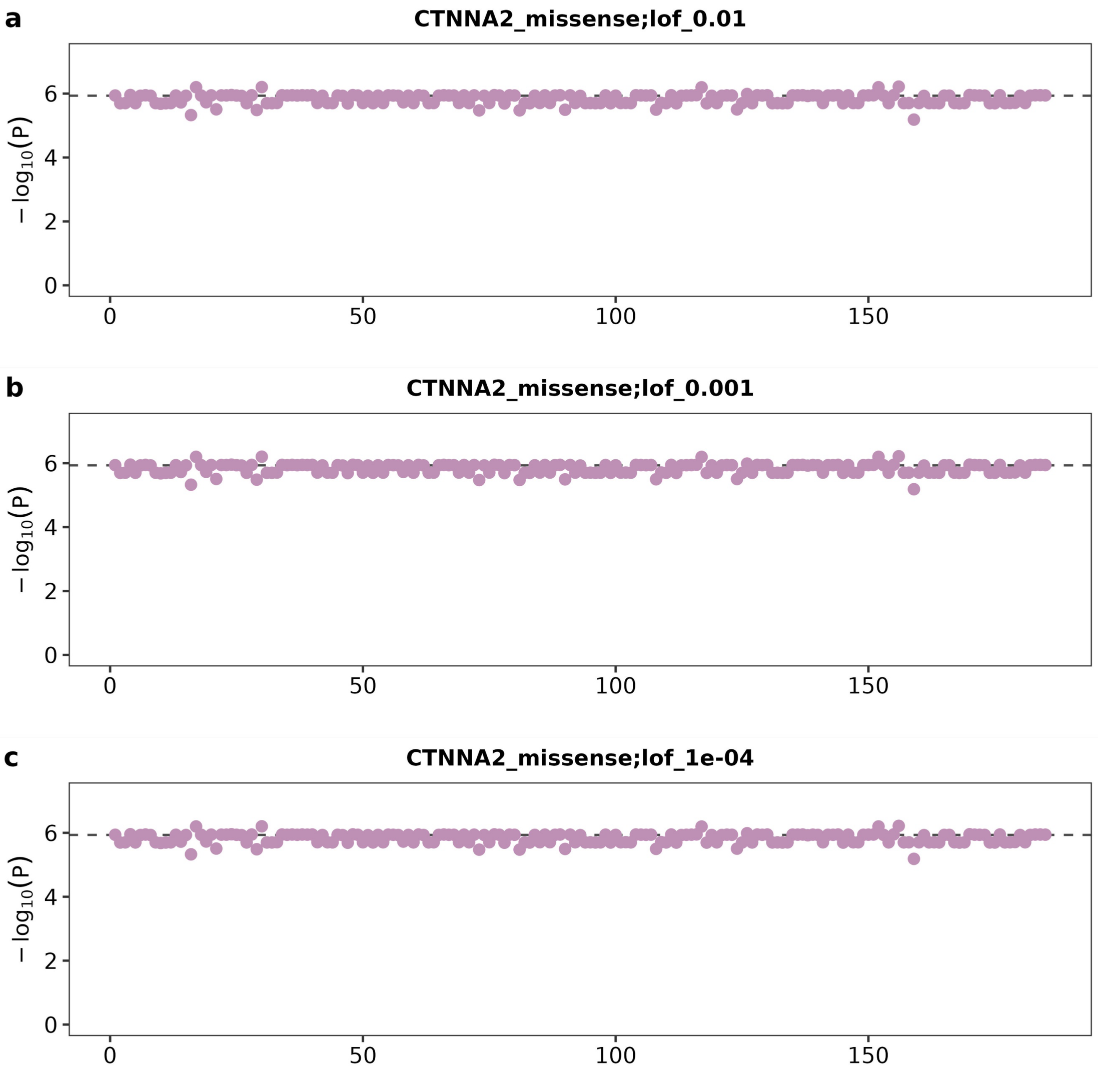

Supplementary Fig. 17 Leave-one-variant-out (LOVO) analysis of the *CTNNA2* gene.

a. Results of the LOVO analysis of the *CTNNA2* gene in the LOF plus missense and max MAF<0.01 group. The *x* axis represents a single variant removed from the gene-based analysis, while the *y* axis represented the $-\log_{10}(P)$ of the association without that given variant. P-values shown are two-sided and unadjusted for multiple testing. b. Results of the LOVO analysis of the *CTNNA2* gene in the LOF plus missense and max MAF<0.001 group. c. Results of the LOVO analysis of the *CTNNA2* gene in the LOF plus missense and max MAF<0.0001 group.

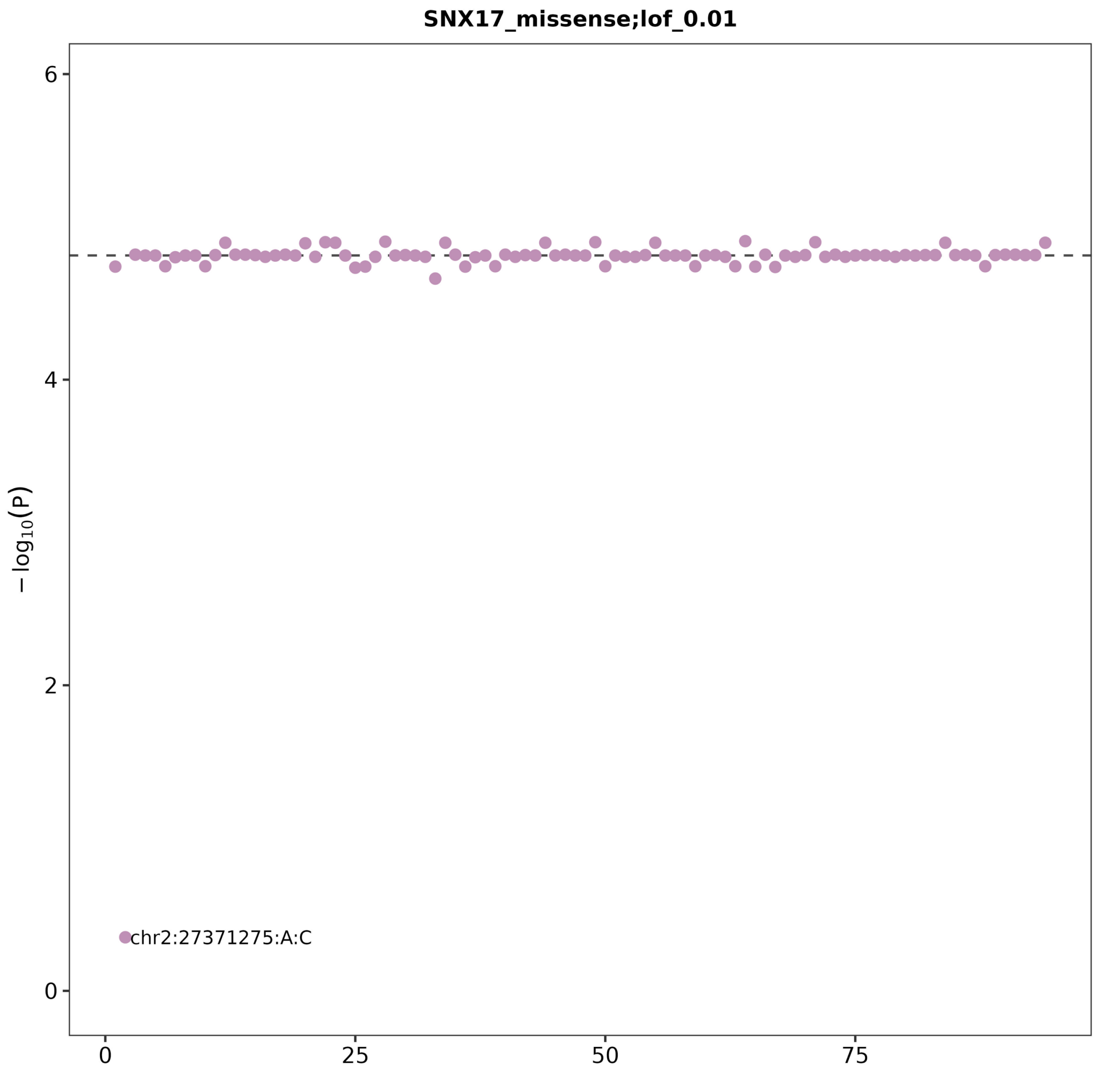

Supplementary Fig. 18 Leave-one-variant-out (LOVO) analysis of the *SNX17* gene.

Results of the LOVO analysis of the *SNX17* gene in the LOF plus missense and max MAF<0.01 group. The *x* axis represents a single variant removed from the gene-based analysis, while the *y* axis represented the $-\log_{10}(P)$ of the association without that given variant. P-values shown are two-sided and unadjusted for multiple testing.

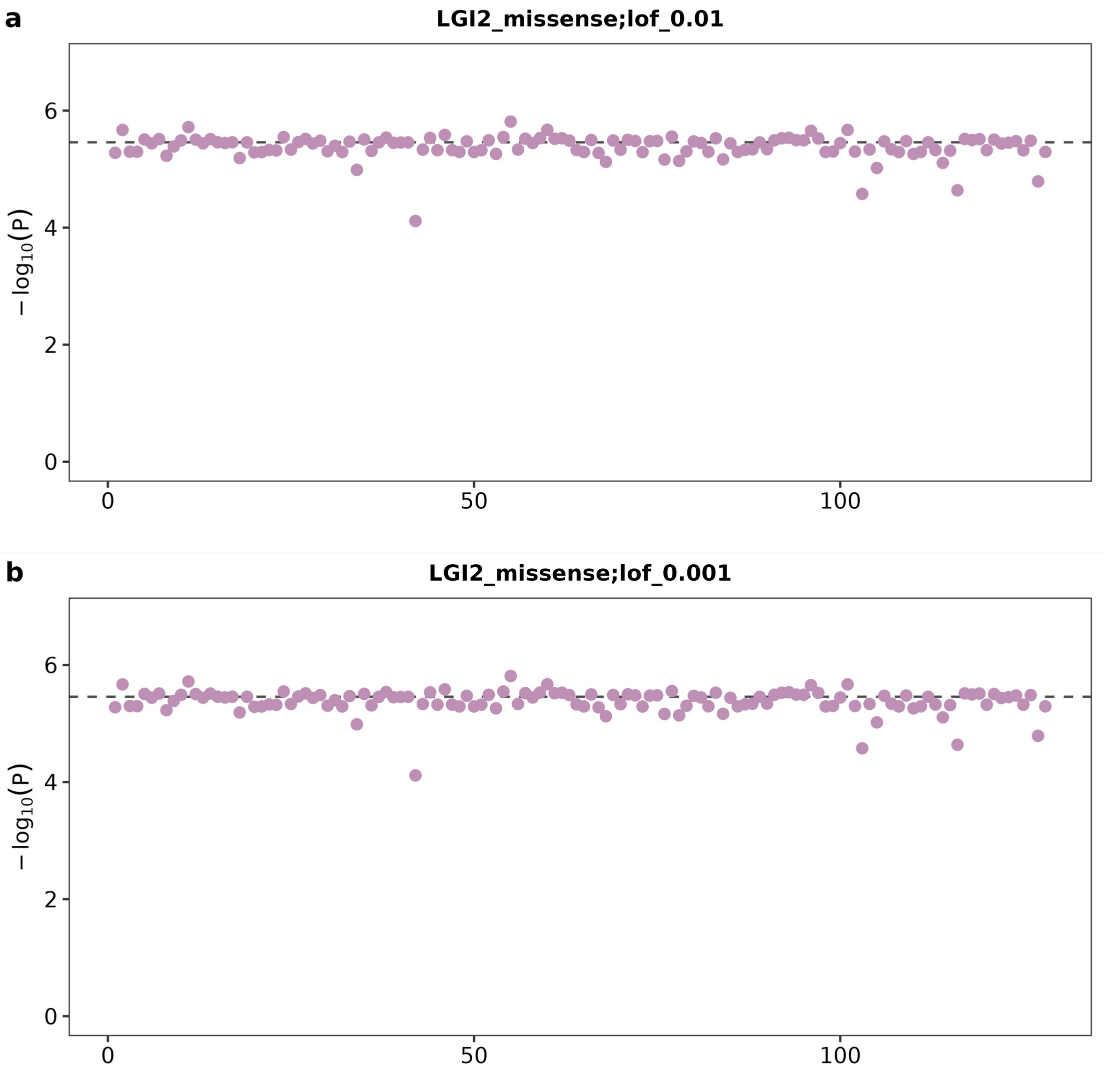

Supplementary Fig. 19 Leave-one-variant-out (LOVO) analysis of the *LGI2* gene.

a. Results of the LOVO analysis of the *LGI2* gene in the LOF plus missense and max MAF<0.01 group. The *x* axis represents a single variant removed from the gene-based analysis, while the *y* axis represented the -$\log_{10}(P)$ of the association without that given variant. P-values shown are two-sided and unadjusted for multiple testing. b. Results of the LOVO analysis of the *LGI2* gene in the LOF plus missense and max MAF<0.001 group.

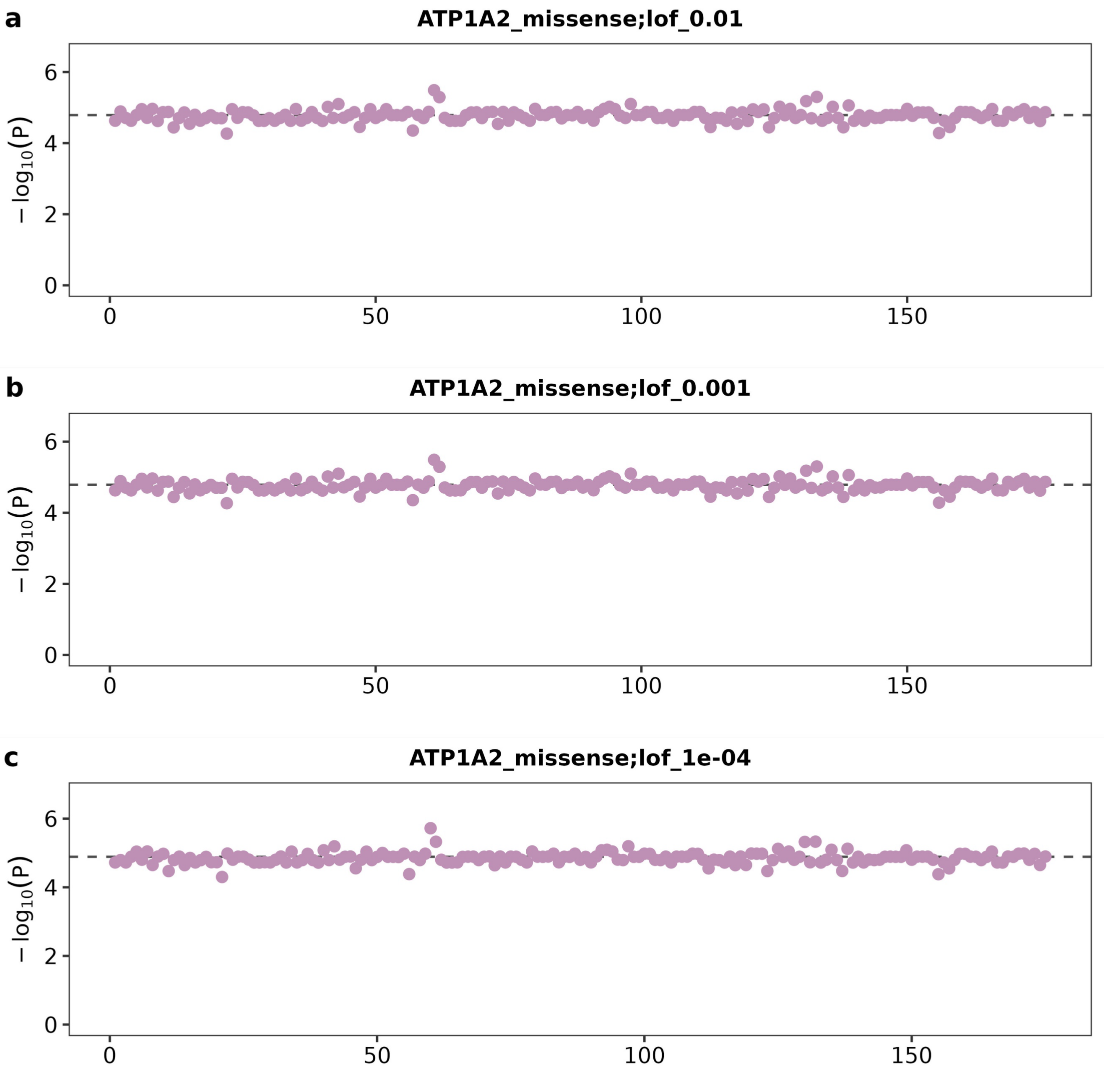

Supplementary Fig. 20 Leave-one-variant-out (LOVO) analysis of the *ATP1A2* gene.

a. Results of the LOVO analysis of the *ATP1A2* gene in the LOF plus missense and max MAF<0.01 group. The *x* axis represents a single variant removed from the gene-based analysis, while the *y* axis represented the -$\log_{10}(P)$ of the association without that given variant. P-values shown are two-sided and unadjusted for multiple testing. b. Results of the LOVO analysis of the *ATP1A2* gene in the LOF plus missense and max MAF<0.001 group. c. Results of the LOVO analysis of the *ATP1A2* gene in the LOF plus missense and max MAF<0.0001 group.

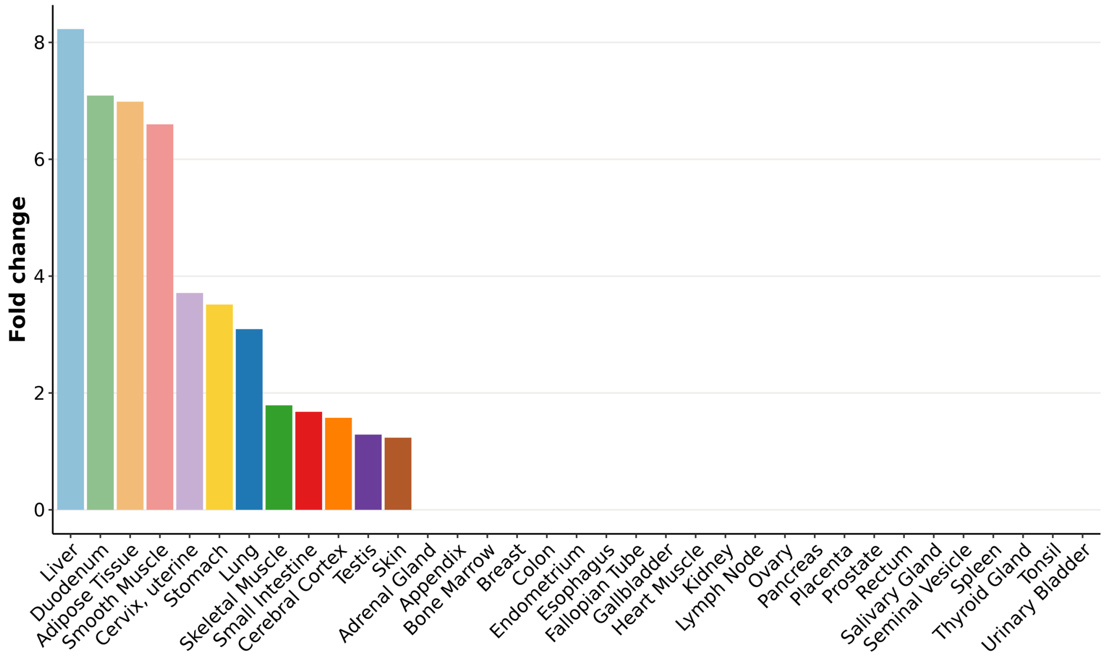

Supplementary Fig. 21 Tissue-specificity analysis of the alcohol consumption genes.

The bar plot showing the tissue-specific gene enrichment. The *x* axis represented the tissues and the *y* axis indicates the fold-change values of the tissue-specific gene enrichment. The color indicates different tissues.

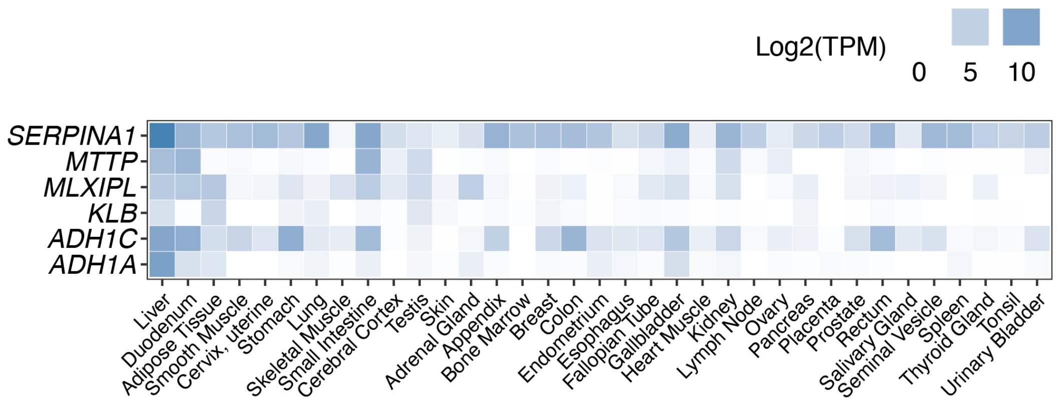

Supplementary Fig. 22 Expression of genes specifically enriched in liver.

The heatmap showing the expression of the liver specific genes across all tissues. The *x* axis represented the tissues and the *y* axis represented the genes. The color represented the quantity of gene expression. Abbreviations: TPM, tissue per million.

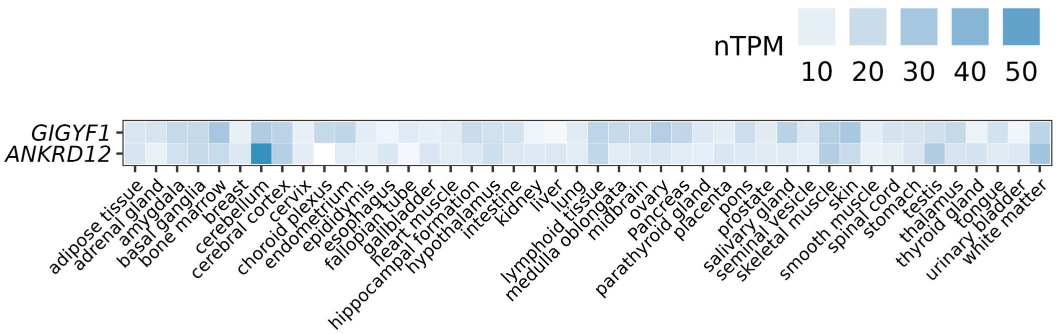

Supplementary Fig. 23 Expression of the *GIGYF1* and *ANKRD12* gene.

The heatmap showing the expression of the two unreported alcohol consumption genes across all tissues. The *x* axis represented the tissues and the *y* axis represented the genes. The color represented the quantity of gene expression. Abbreviations: nTPM, normalised tissue per millon.

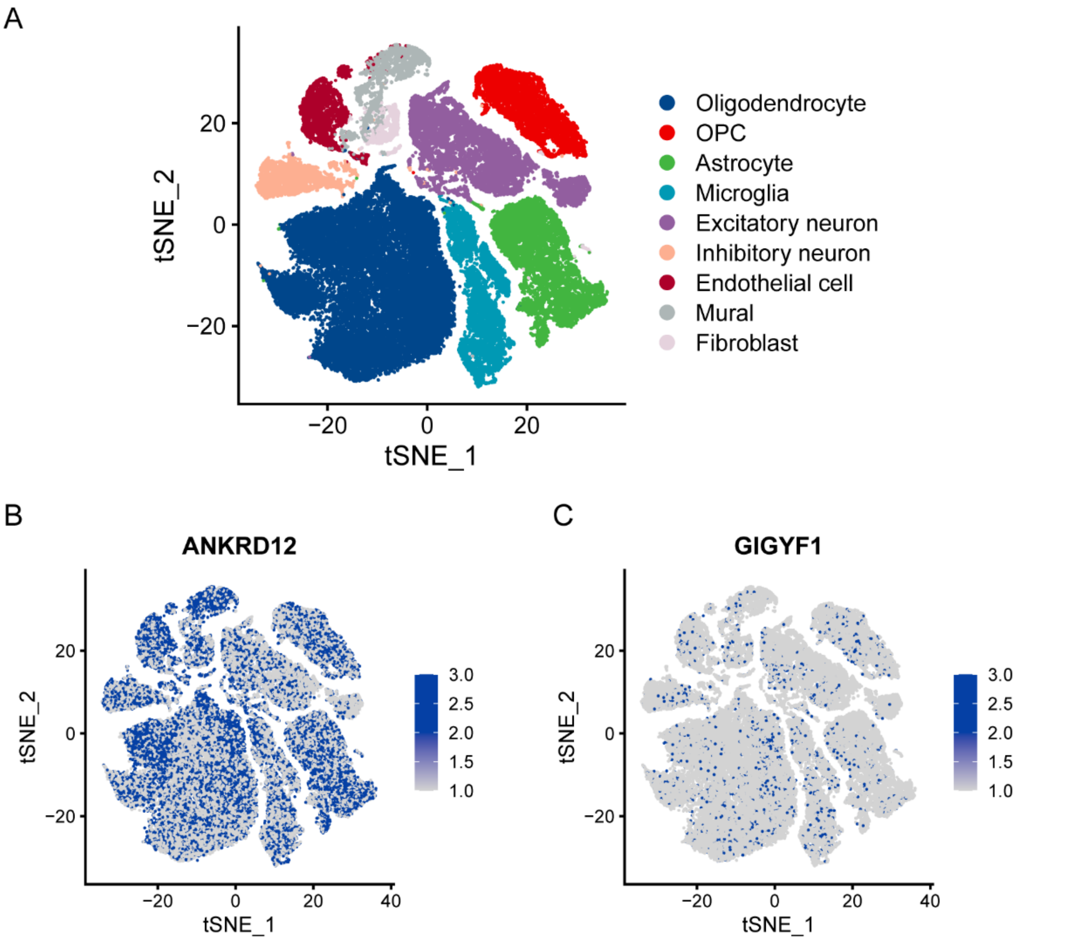

Supplementary Fig. 24 Cell-type expression of the *ANKRD12* and *GIGYF1* gene in the brain.

A. The t–Stochastic Neighbourhood Embedding (tSNE) plot showing different cell types within the brain. The color of the dots indicates the cell type. B. The feature plot showing the expression level of *ANKRD12* in different cell types within brain. C. The feature plot showing the expression level of *GIGYF1* in different cell types within brain. Abbreviations: TPM: transcripts per million.

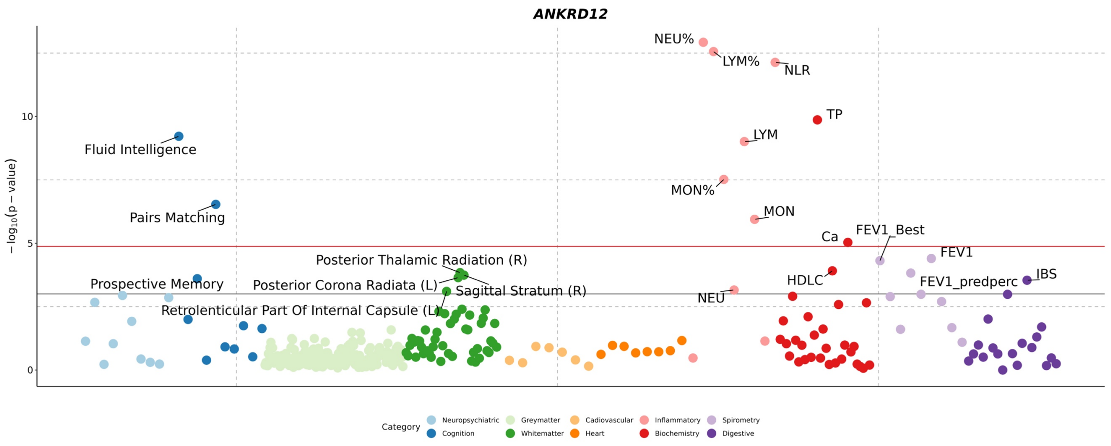

Supplementary Fig. 25 PheWAS results of *ANKRD12*.

The x axis represented the phenotype categories and the y axis represented the -$\log_{10}(P)$. P-values shown are two-sided and unadjusted for multiple testing. The red horizontal line indicates the threshold for significant association (P <0.05/316/12=1.32×10^{-5}). The grey horizontal line denotes the threshold for a significant association to a lesser extent (P <0.001). Abbreviations: PheWAS, phenome-wide association studies; NEU%, Neutrophill Percentage; LYM%, Lymphocyte Percentage; NLR, Neutrophill Lymphocyte Ratio; TP, Total Protein; Fluid Intelligence, Fluid Intelligence Score; LYM, Lymphocyte Count; MON%, Monocyte Percentage; Pairs Matching, Number Of Incorrect Matches In Round; MON, Monocyte Count; Ca, Calcium; FEV1, Forced Expiratory Volume In 1-Second (Fev1); FEV1_Best, Forced Expiratory Volume In 1-Second (Fev1), Best Measure; HDLC, Hdl Cholesterol; Posterior Thalamic Radiation (R), Posterior Thalamic Radiation (R); FEV1_predperc, Forced Expiratory Volume In 1-Second (Fev1), Predicted Percentage; Sagittal Stratum (R), Sagittal Stratum (R); Posterior Corona Radiata (L), Posterior Corona Radiata (L); Prospective Memory, Prospective Memory Result; IBS, Inflammatory Bowel Disease; NEU, Neutrophill Count; Retrolenticular

Part Of Internal Capsule (L), Retrolenticular Part Of Internal Capsule (L).

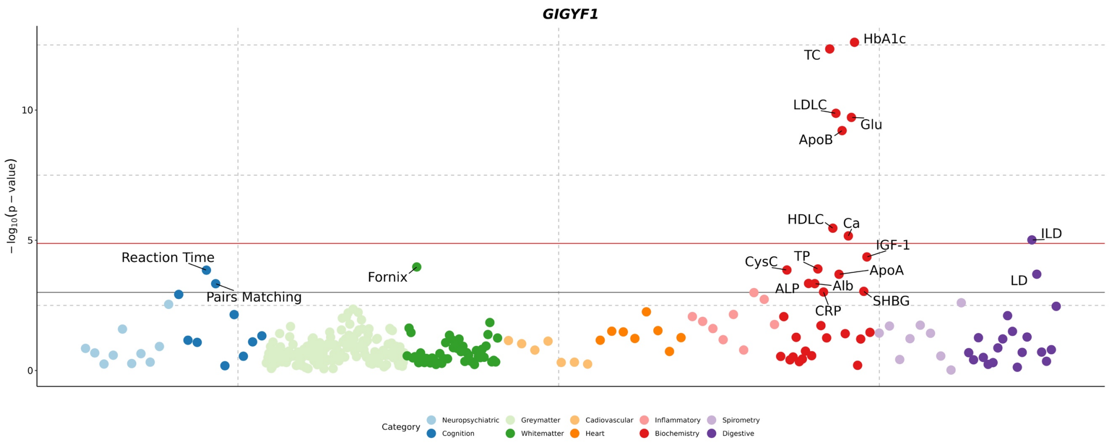

Supplementary Fig. 26 PheWAS results of *GIGYF1*.

The *x* axis represented the phenotype categories and the *y* axis represented the -log$_{10}$(P). P-values shown are two-sided and unadjusted for multiple testing. The red horizontal line indicates the threshold for significant association (P <0.05/316/12=1.32×10^{-5}). The grey horizontal line denotes the threshold for a significant association to a lesser extent (P <0.001). Abbreviations: PheWAS, phenome-wide association studies; HbA1c, Glycated Haemoglobin (Hba1C); TC, Cholesterol; LDLC, Ldl Direct; Glu, Glucose; ApoB, Apolipoprotein B; HDLC, Hdl Cholesterol; Ca, Calcium; ILD, Inflammatory Liver Disease; IGF-1, IGF1; Fornix, Fornix; TP, Total Protein; CysC, Cystatin C; Reaction Time, Mean Time To Correctly Identify Matches; LD, Liver Disease; ApoA, Apolipoprotein A; ALP, Alkaline Phosphatase; Alb, Albumin; Pairs Matching, Number Of Incorrect Matches In Round; SHBG, Sex Hormone-Binding Globulin, Shbg; CRP, C-reactive protein.

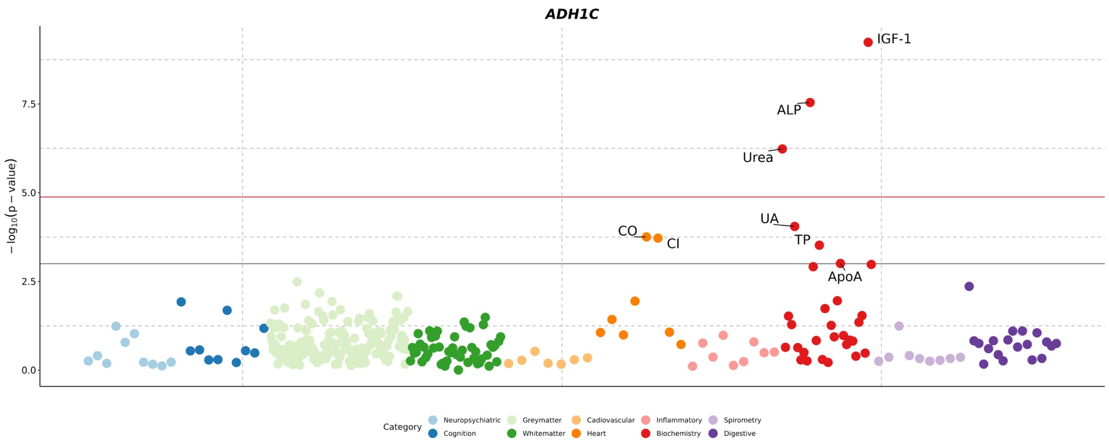

Supplementary Fig. 27 PheWAS results of *ADH1C*.

The *x* axis represented the phenotype categories and the *y* axis represented the $-\log_{10}(P)$. P-values shown are two-sided and unadjusted for multiple testing. The red horizontal line indicates the threshold for significant association ($P < 0.05/316/12 = 1.32\times10^{-5}$). The grey horizontal line denotes the threshold for a significant association to a lesser extent ($P < 0.001$). Abbreviations: PheWAS, phenome-wide association studies; IGF-1, IGF1; ALP, Alkaline Phosphatase; Urea, Urea; UA, Urate; CO, Cardiac Output; CI, Cardiac Index; TP, Total Protein; ApoA, Apolipoprotein A.

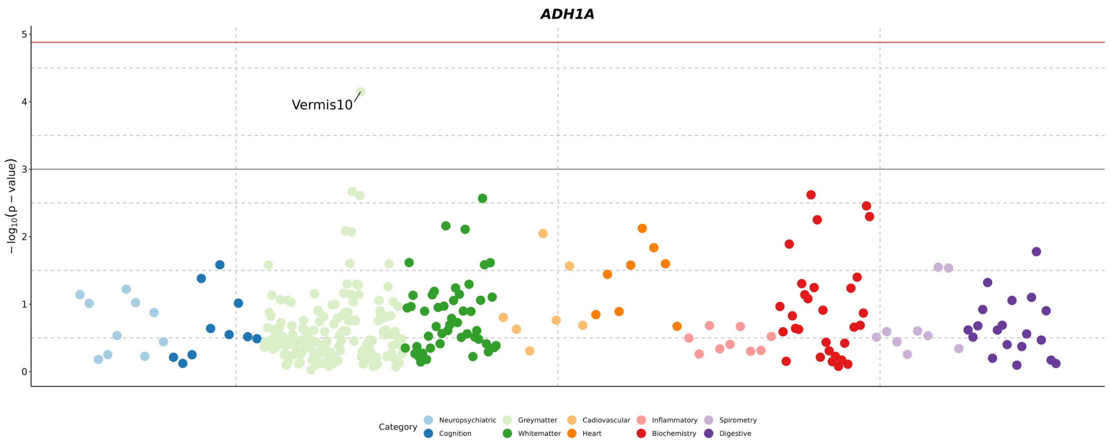

Supplementary Fig. 28 PheWAS results of *ADH1A*.

The *x* axis represented the phenotype categories and the *y* axis represented the -$\log_{10}(P)$. P-values shown are two-sided and unadjusted for multiple testing. The red horizontal line indicates the threshold for significant association (P <0.05/316/12=1.32×10^{-5}). The grey horizontal line denotes the threshold for a significant association to a lesser extent (P <0.001). Abbreviations: PheWAS, phenome-wide association studies.

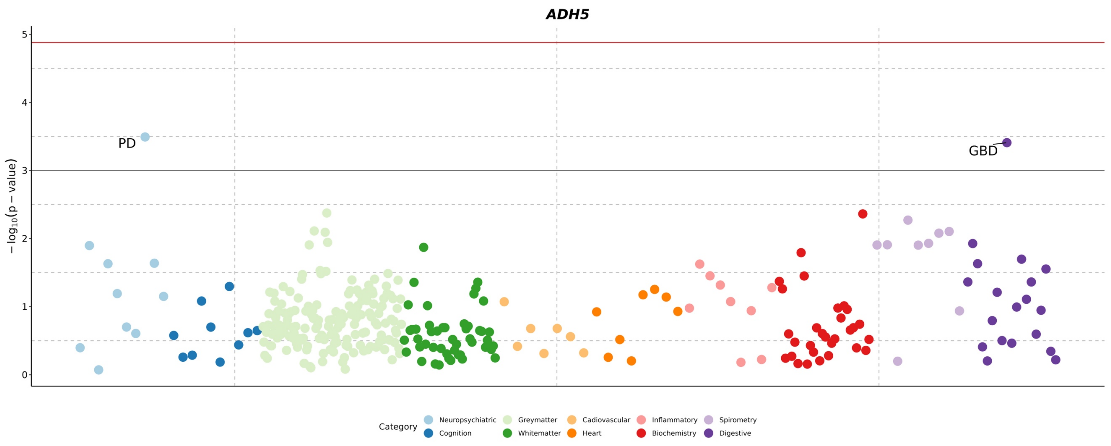

Supplementary Fig. 29 PheWAS results of *ADH5*.

The *x* axis represented the phenotype categories and the *y* axis represented the -$\log_{10}(P)$. P-values shown are two-sided and unadjusted for multiple testing. The red horizontal line indicates the threshold for significant association (P <0.05/316/12=1.32×10^{-5}). The grey horizontal line denotes the threshold for a significant association to a lesser extent (P <0.001). Abbreviations: PheWAS, phenome-wide association studies; PD, Parkinson's Disease; GBD, Gallbladder Disease.

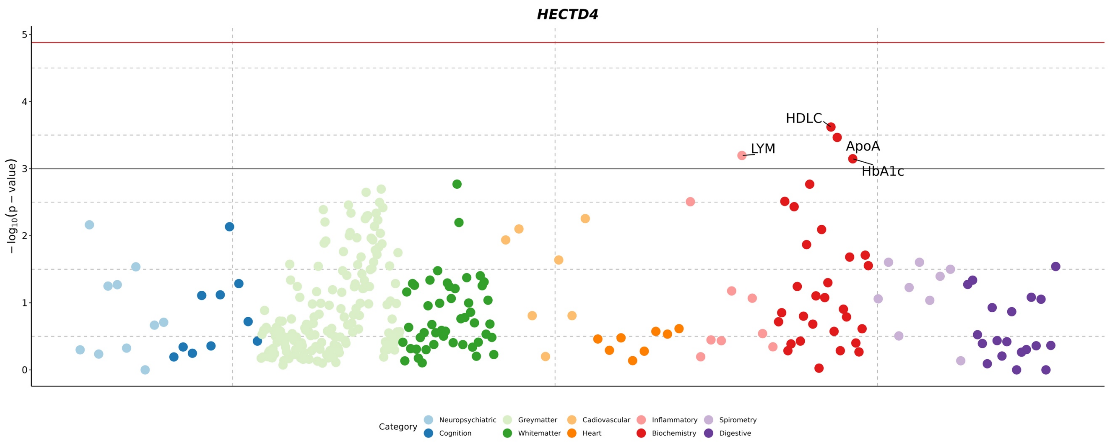

Supplementary Fig. 30 PheWAS results of *HECTD4*.

The x axis represented the phenotype categories and the y axis represented the -$\log_{10}(P)$. P-values shown are two-sided and unadjusted for multiple testing. The red horizontal line indicates the threshold for significant association ($P < 0.05/316/12 = 1.32 \times 10^{-5}$). The grey horizontal line denotes the threshold for a significant association to a lesser extent ($P < 0.001$). Abbreviations: PheWAS, phenome-wide association studies; HDLC, Hdl Cholesterol; ApoA, Apolipoprotein A; LYM, Lymphocyte Count; HbA1c, Glycated Haemoglobin (Hba1C).

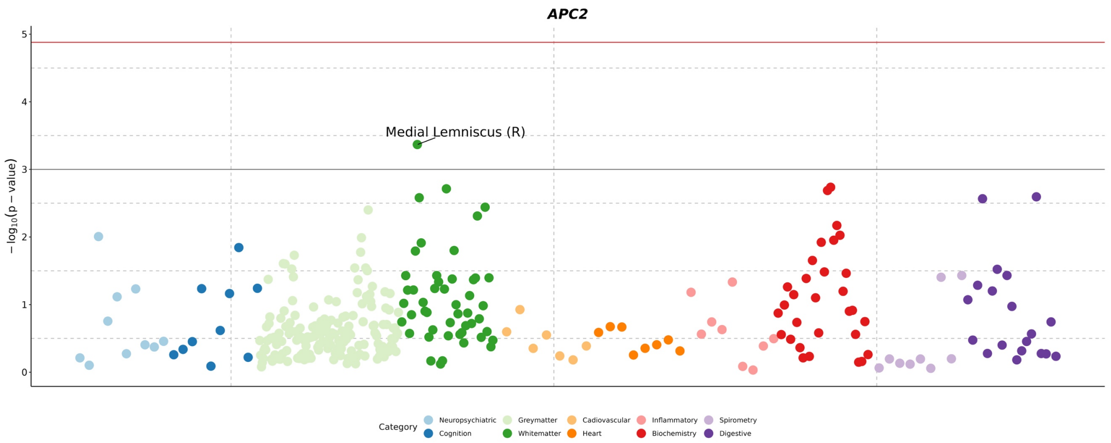

Supplementary Fig. 31 PheWAS results of *APC2*.

The *x* axis represented the phenotype categories and the *y* axis represented the -$\log_{10}(P)$. P-values shown are two-sided and unadjusted for multiple testing. The red horizontal line indicates the threshold for significant association (P <0.05/316/12=1.32×10^{-5}). The grey horizontal line denotes the threshold for a significant association to a lesser extent (P <0.001). Abbreviations: PheWAS, phenome-wide association studies.

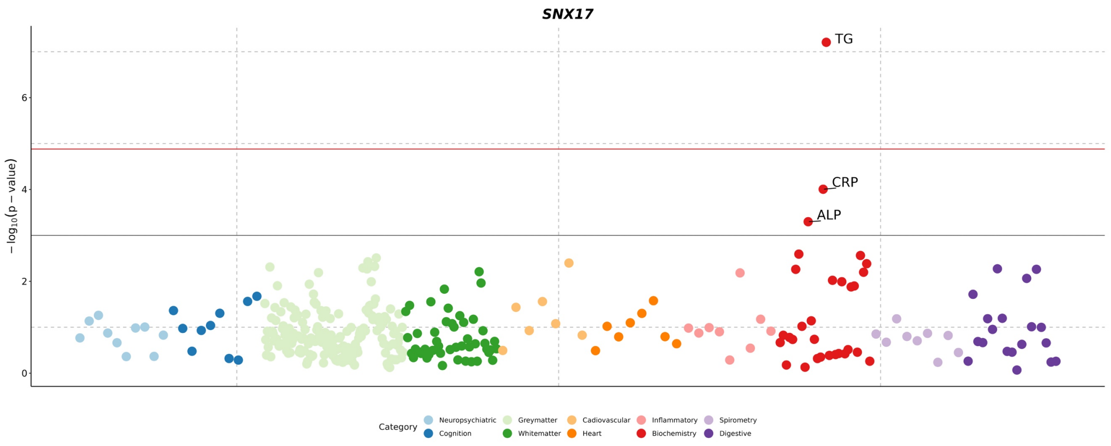

Supplementary Fig. 32 PheWAS results of *SNX17*.

The x axis represented the phenotype categories and the y axis represented the $-\log_{10}(P)$. P-values shown are two-sided and unadjusted for multiple testing. The red horizontal line indicates the threshold for significant association (P <0.05/316/12=1.32×10^{-5}). The grey horizontal line denotes the threshold for a significant association to a lesser extent (P <0.001). Abbreviations: PheWAS, phenome-wide association studies; TG, Triglycerides; CRP, C-Reactive Protein; ALP, Alkaline Phosphatase.

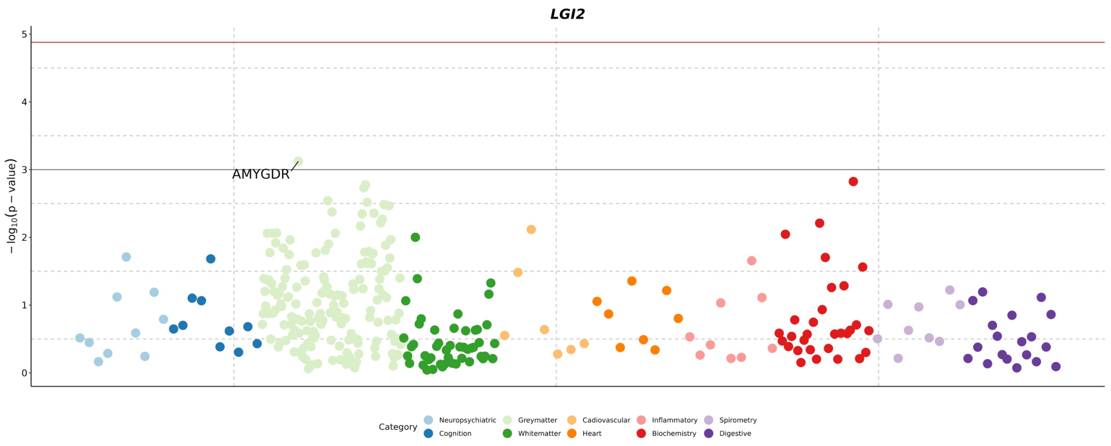

Supplementary Fig. 33 PheWAS results of *LGI2*.

The *x* axis represented the phenotype categories and the *y* axis represented the $-\log_{10}(P)$. P-values shown are two-sided and unadjusted for multiple testing. The red horizontal line indicates the threshold for significant association ($P < 0.05/316/12 = 1.32\times10^{-5}$). The grey horizontal line denotes the threshold for a significant association to a lesser extent ($P < 0.001$). Abbreviations: PheWAS, phenome-wide association studies; AMYGDR, Amygdala_R.

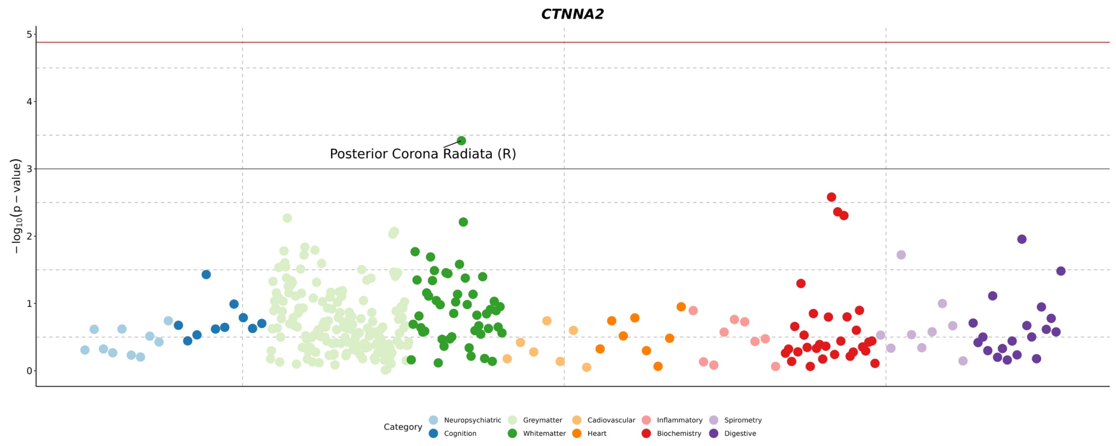

Supplementary Fig. 34 PheWAS results of *CTNNA2*.

The *x* axis represented the phenotype categories and the *y* axis represented the -$\log_{10}(P)$. P-values shown are two-sided and unadjusted for multiple testing. The red horizontal line indicates the threshold for significant association ($P < 0.05/316/12 = 1.32\times10^{-5}$). The grey horizontal line denotes the threshold for a significant association to a lesser extent ($P < 0.001$).

Abbreviations: PheWAS, phenome-wide association studies.

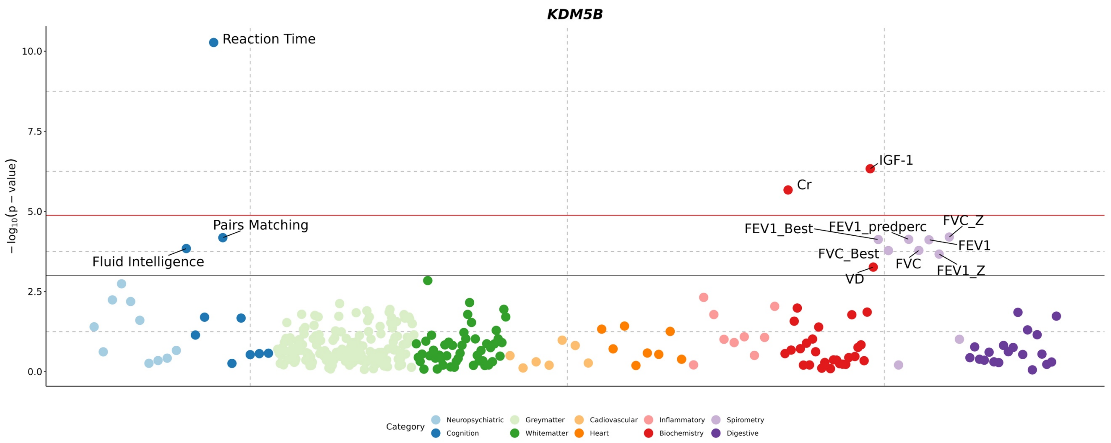

Supplementary Fig. 35 PheWAS results of *KDM5B*.

The x axis represented the phenotype categories and the y axis represented the $-\log_{10}(P)$. P-values shown are two-sided and unadjusted for multiple testing. The red horizontal line indicates the threshold for significant association ($P < 0.05/316/12 = 1.32 \times 10^{-5}$). The grey horizontal line denotes the threshold for a significant association to a lesser extent ($P < 0.001$). Abbreviations: PheWAS, phenome-wide association studies; Reaction Time, Mean Time To Correctly Identify Matches; IGF-1, IGF1; Cr, Creatinine; FVC_Z, Forced Vital Capacity (Fvc) Z-Score; Pairs Matching, Number Of Incorrect Matches In Round; FEV1_predperc, Forced Expiratory Volume In 1-Second (Fev1), Predicted Percentage; FEV1_Best, Forced Expiratory Volume In 1-Second (Fev1), Best Measure; FEV1, Forced Expiratory Volume In 1-Second (Fev1); Fluid Intelligence, Fluid Intelligence Score; FVC, Forced Vital Capacity (Fvc); FVC_Best, Forced Vital Capacity (Fvc), Best Measure; FEV1_Z, Forced Expiratory Volume In 1-Second (Fev1) Z-Score; VD, Vitamin D.

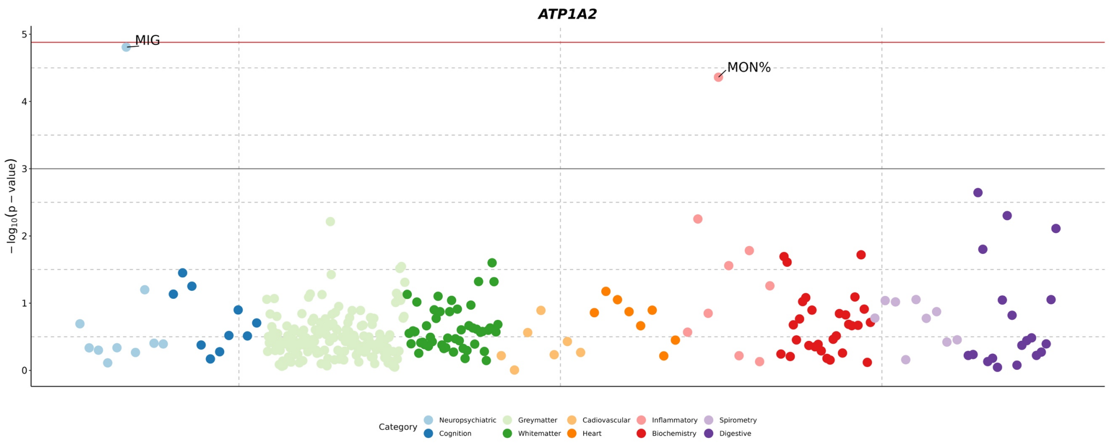

Supplementary Fig. 36 PheWAS results of *ATP1A2*.

The *x* axis represented the phenotype categories and the *y* axis represented the -$\log_{10}(P)$. P-values shown are two-sided and unadjusted for multiple testing. The red horizontal line indicates the threshold for significant association (P <0.05/316/12=1.32×10^{-5}). The grey horizontal line denotes the threshold for a significant association to a lesser extent (P <0.001). Abbreviations: PheWAS, phenome-wide association studies; MIG, Migraine; MON%, Monocyte Percentage.

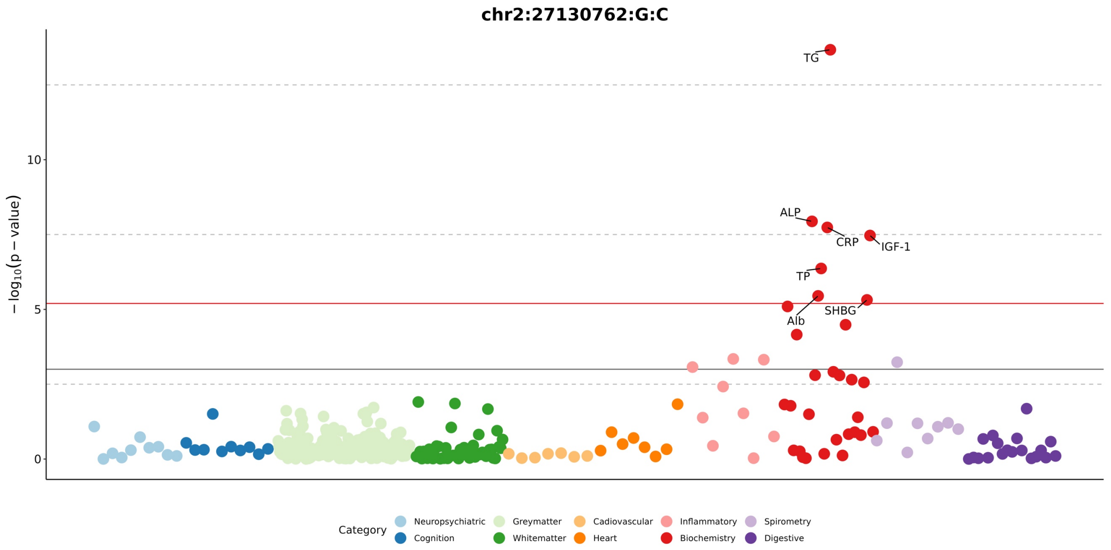

Supplementary Fig. 37 PheWAS results of variant chr2:27130762:G:C.

The x axis represented the phenotype categories and the y axis represented the -$\log_{10}(P)$. P-values shown are two-sided and unadjusted for multiple testing. The red horizontal line indicates the threshold for significant association (P <0.05/316/25=6.33×10^{-6}). The grey horizontal line denotes the threshold for a significant association to a lesser extent (P <0.001). Abbreviations: PheWAS, phenome-wide association studies; TG, Triglycerides; ALP, Alkaline Phosphatase; CRP, C-Reactive Protein; IGF-1, IGF1; TP, Total Protein; Alb, Albumin; SHBG, Sex Hormone-Binding Globulin, Shbg.

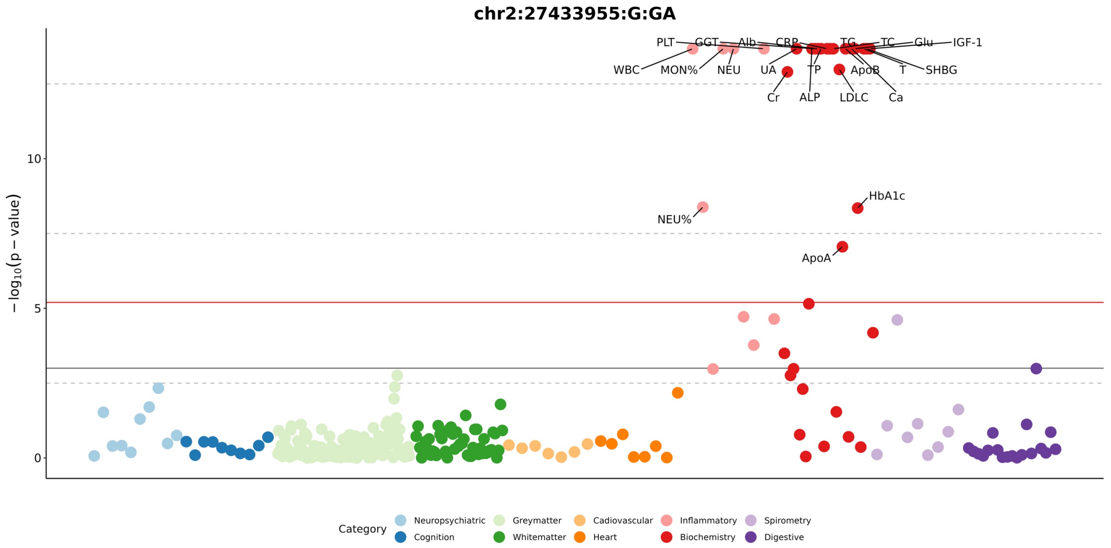

Supplementary Fig. 38 PheWAS results of variant chr2:27433955:G:GA.

The *x* axis represented the phenotype categories and the *y* axis represented the $-\log_{10}(P)$. P-values shown are two-sided and unadjusted for multiple testing. Values above $-\log_{10}(2.125\times10^{-14})$ are capped. The red horizontal line indicates the threshold for significant association (P <0.05/316/25=6.33×10^{-6}). The grey horizontal line denotes the threshold for a significant association to a lesser extent (P <0.001). Abbreviations: PheWAS, phenome-wide association studies; TG, Triglycerides; CRP, C-Reactive Protein; SHBG, Sex Hormone-Binding Globulin, Shbg; Alb, Albumin; GGT, Gamma Glutamyltransferase; UA, Urate; ALP, Alkaline Phosphatase; MON%, Monocyte Percentage; Ca, Calcium; IGF-1, IGF1; TP, Total Protein; PLT, Platelet Count; ApoB, Apolipoprotein B; T, Testosterone; Glu, Glucose; NEU, Neutrophill Count; TC, Cholesterol; WBC, White Blood Cell (Leukocyte) Count; LDLC, Ldl Direct; Cr, Creatinine; NEU%, Neutrophill Percentage; HbA1c, Glycated Haemoglobin (Hba1C); ApoA, Apolipoprotein A.

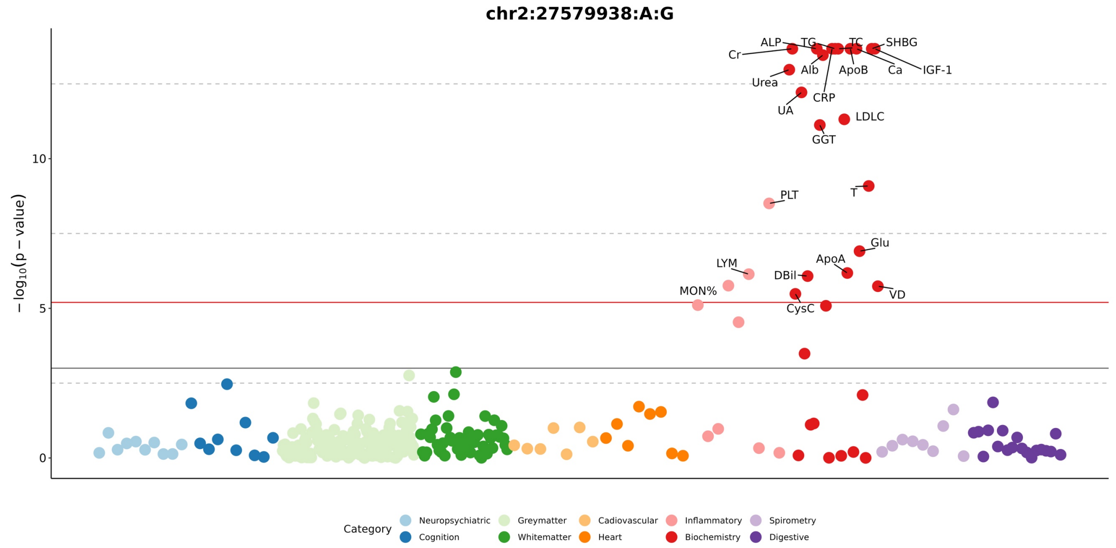

Supplementary Fig. 39 PheWAS results of variant chr2:27579938:A:G.

The x axis represented the phenotype categories and the y axis represented the $-\log_{10}(P)$. P-values shown are two-sided and unadjusted for multiple testing. The red horizontal line indicates the threshold for significant association ($P < 0.05/316/25 = 6.33\times10^{-6}$). The grey horizontal line denotes the threshold for a significant association to a lesser extent ($P < 0.001$). Values above $-\log_{10}(2.125\times10^{-14})$ are capped. Abbreviations: PheWAS, phenome-wide association studies; TG, Triglycerides; SHBG, Sex Hormone-Binding Globulin, Shbg; IGF-1, IGF1; CRP, C-Reactive Protein; ApoB, Apolipoprotein B; Cr, Creatinine; TC, Cholesterol; ALP, Alkaline Phosphatase; Ca, Calcium; Alb, Albumin; Urea, Urea; UA, Urate; LDLC, Ldl Direct; GGT, Gamma Glutamyltransferase; T, Testosterone; PLT, Platelet Count; Glu, Glucose; ApoA, Apolipoprotein A; LYM, Lymphocyte Count; DBil, Direct Bilirubin; MON%, Monocyte Percentage; VD, Vitamin D; CysC, Cystatin C.

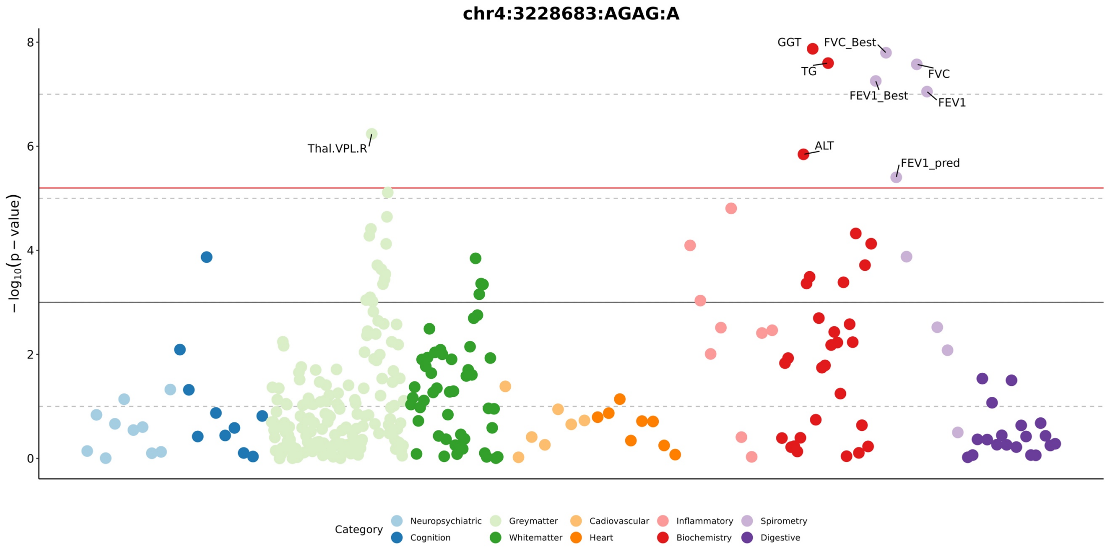

Supplementary Fig. 40 PheWAS results of variant chr4:3228683:AGAG:A.

The x axis represented the phenotype categories and the y axis represented the $-\log_{10}(P)$. P-values shown are two-sided and unadjusted for multiple testing. The red horizontal line indicates the threshold for significant association ($P < 0.05/316/25 = 6.33 \times 10^{-6}$). The grey horizontal line denotes the threshold for a significant association to a lesser extent ($P < 0.001$). Abbreviations: PheWAS, phenome-wide association studies; GGT, Gamma Glutamyltransferase; FVC_Best, Forced Vital Capacity (Fvc), Best Measure; TG, Triglycerides; FVC, Forced Vital Capacity (Fvc); FEV1_Best, Forced Expiratory Volume In 1-Second (Fev1), Best Measure; FEV1, Forced Expiratory Volume In 1-Second (Fev1); Thal.VPL.R, Thal_Vpl_R; ALT, Alanine Aminotransferase; FEV1_pred, Forced Expiratory Volume In 1-Second (Fev1), Predicted.

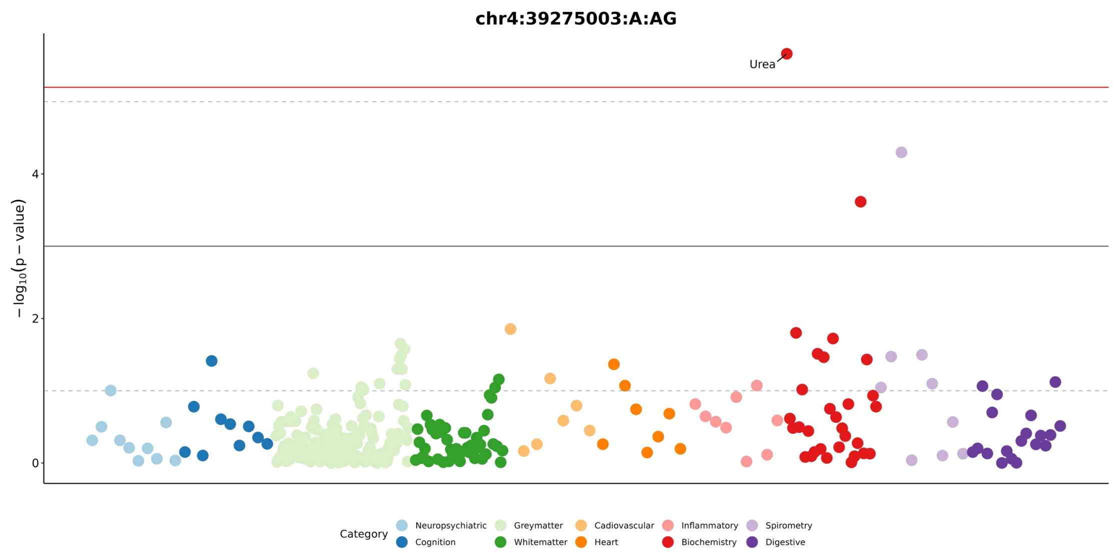

Supplementary Fig. 41 PheWAS results of variant chr4:39275003:A:AG.

The *x* axis represented the phenotype categories and the *y* axis represented the $-\log_{10}(P)$. P-values shown are two-sided and unadjusted for multiple testing. The red horizontal line indicates the threshold for significant association ($P < 0.05/316/25 = 6.33\times10^{-6}$). The grey horizontal line denotes the threshold for a significant association to a lesser extent ($P < 0.001$). Abbreviations: PheWAS, phenome-wide association studies; Urea, Urea.

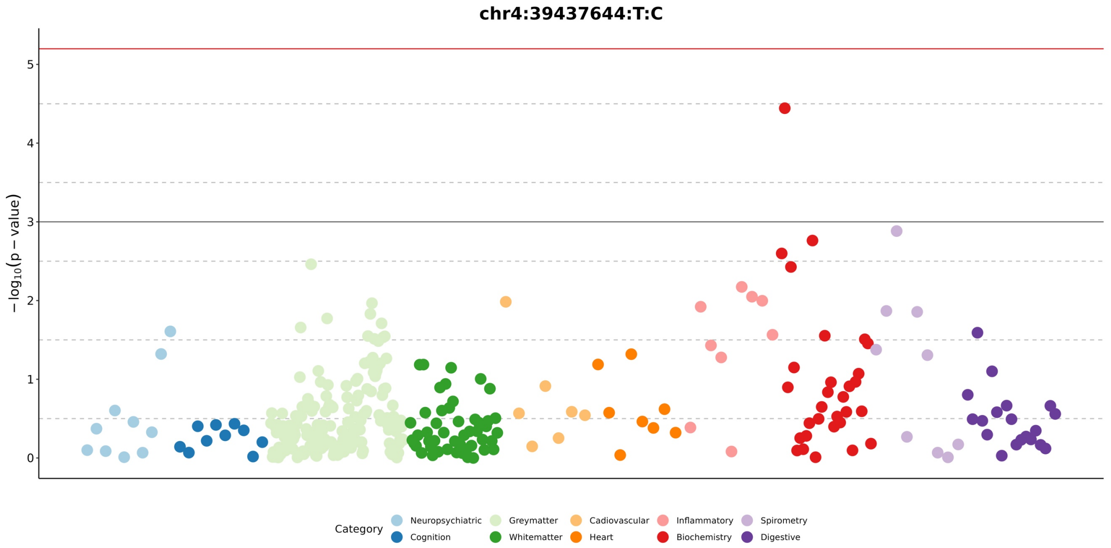

Supplementary Fig. 42 PheWAS results of variant chr4:39437644:T:C.

The x axis represented the phenotype categories and the y axis represented the $-\log_{10}(P)$. P-values shown are two-sided and unadjusted for multiple testing. The red horizontal line indicates the threshold for significant association ($P < 0.05/316/25 = 6.33\times10^{-6}$). The grey horizontal line denotes the threshold for a significant association to a lesser extent ($P < 0.001$). Abbreviations: PheWAS, phenome-wide association studies.

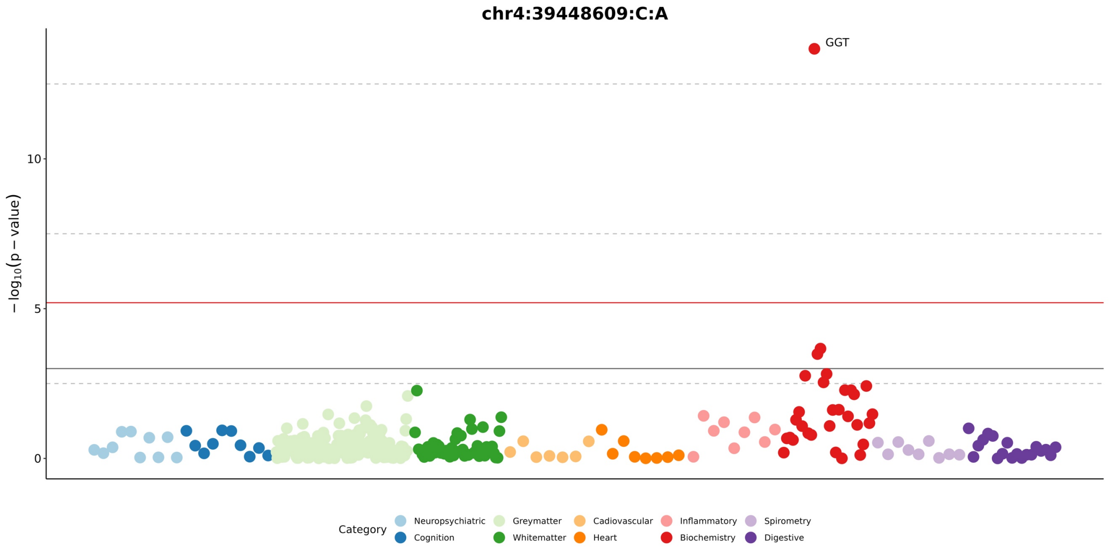

Supplementary Fig. 43 PheWAS results of variant chr4:39448609:C:A.

The *x* axis represented the phenotype categories and the *y* axis represented the -$\log_{10}(P)$. P-values shown are two-sided and unadjusted for multiple testing. The red horizontal line indicates the threshold for significant association (P <0.05/316/25=6.33×10^{-6}). The grey horizontal line denotes the threshold for a significant association to a lesser extent (P <0.001). Values above -$\log_{10}$ (2.125×10^{-14}) are capped. Abbreviations: PheWAS, phenome-wide association studies; GGT, Gamma Glutamyltransferase.

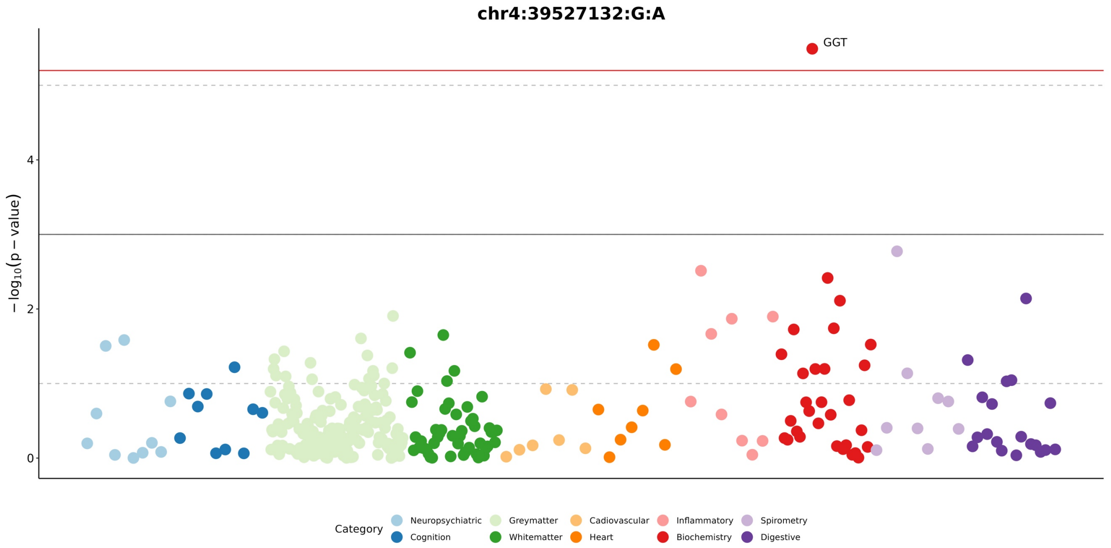

Supplementary Fig. 44 PheWAS results of variant chr4:39527132:G:A.

The *x* axis represented the phenotype categories and the *y* axis represented the $-\log_{10}(P)$. P-values shown are two-sided and unadjusted for multiple testing. The red horizontal line indicates the threshold for significant association ($P < 0.05/316/25 = 6.33 \times 10^{-6}$). The grey horizontal line denotes the threshold for a significant association to a lesser extent ($P < 0.001$). Abbreviations: PheWAS, phenome-wide association studies; GGT, Gamma Glutamyltransferase.

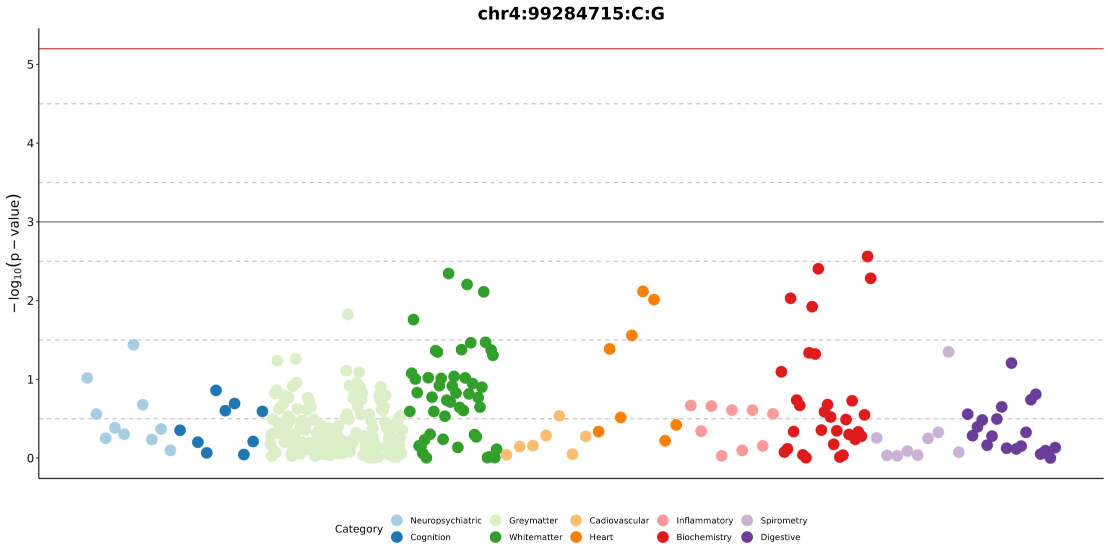

Supplementary Fig. 45 PheWAS results of variant chr4:99284715:C:G.

The *x* axis represented the phenotype categories and the *y* axis represented the $-\log_{10}(P)$. P-values shown are two-sided and unadjusted for multiple testing. The red horizontal line indicates the threshold for significant association (P <0.05/316/25=6.33×10^{-6}). The grey horizontal line denotes the threshold for a significant association to a lesser extent (P <0.001). Abbreviations: PheWAS, phenome-wide association studies.

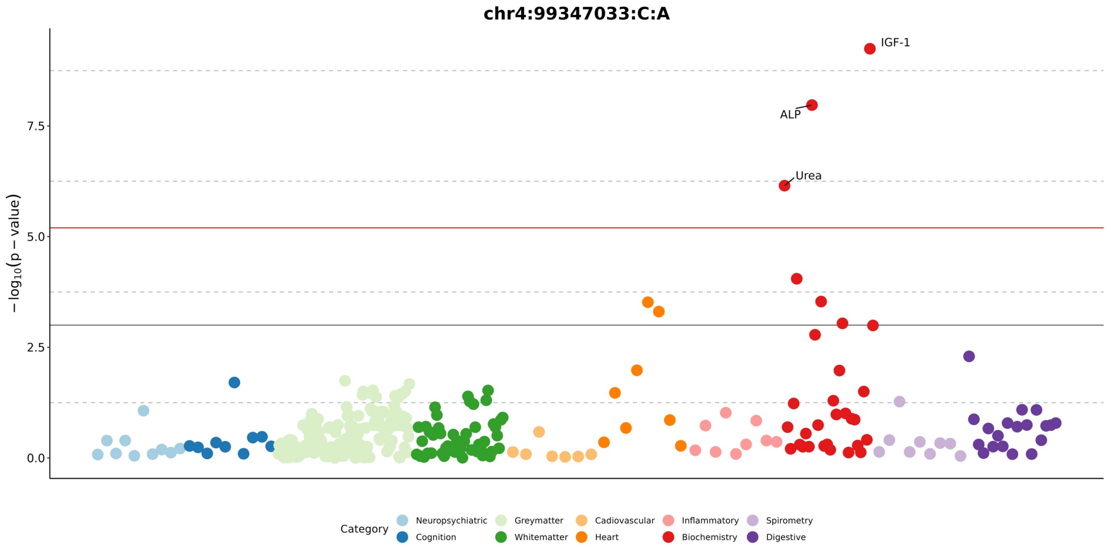

Supplementary Fig. 46 PheWAS results of variant chr4:99347033:C:A.

The *x* axis represented the phenotype categories and the *y* axis represented the -$\log_{10}(P)$. P-values shown are two-sided and unadjusted for multiple testing. The red horizontal line indicates the threshold for significant association (P <0.05/316/25=6.33×10^{-6}). The grey horizontal line denotes the threshold for a significant association to a lesser extent (P <0.001). Abbreviations: PheWAS, phenome-wide association studies; IGF-1, IGF1; ALP, Alkaline Phosphatase .

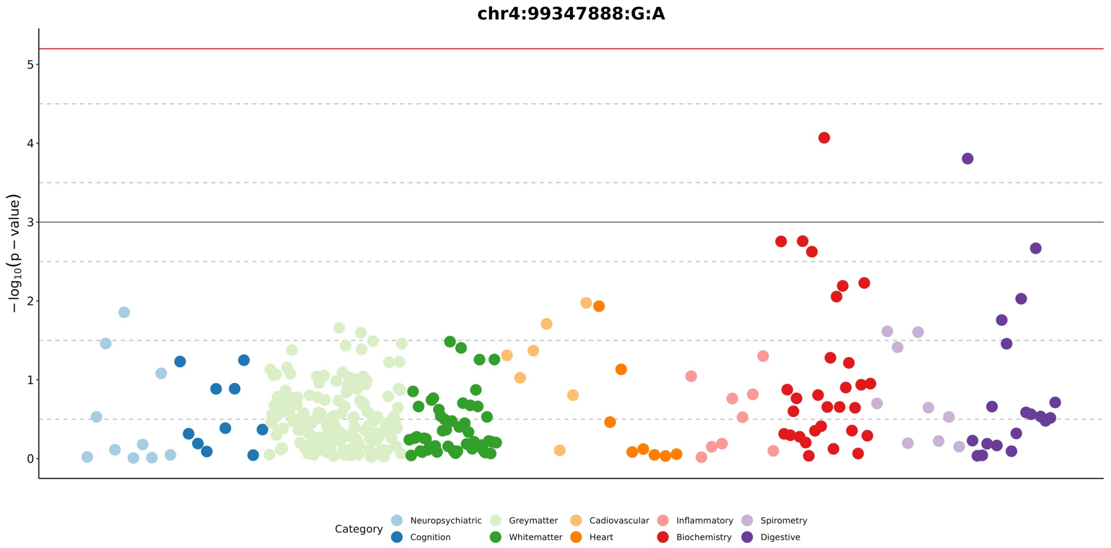

Supplementary Fig. 47 PheWAS results of variant chr4:99347888:G:A.

The *x* axis represented the phenotype categories and the *y* axis represented the -$\log_{10}(P)$. P-values shown are two-sided and unadjusted for multiple testing. The red horizontal line indicates the threshold for significant association (P <0.05/316/25=6.33×10^{-6}). The grey horizontal line denotes the threshold for a significant association to a lesser extent (P <0.001). Abbreviations: PheWAS, phenome-wide association studies.

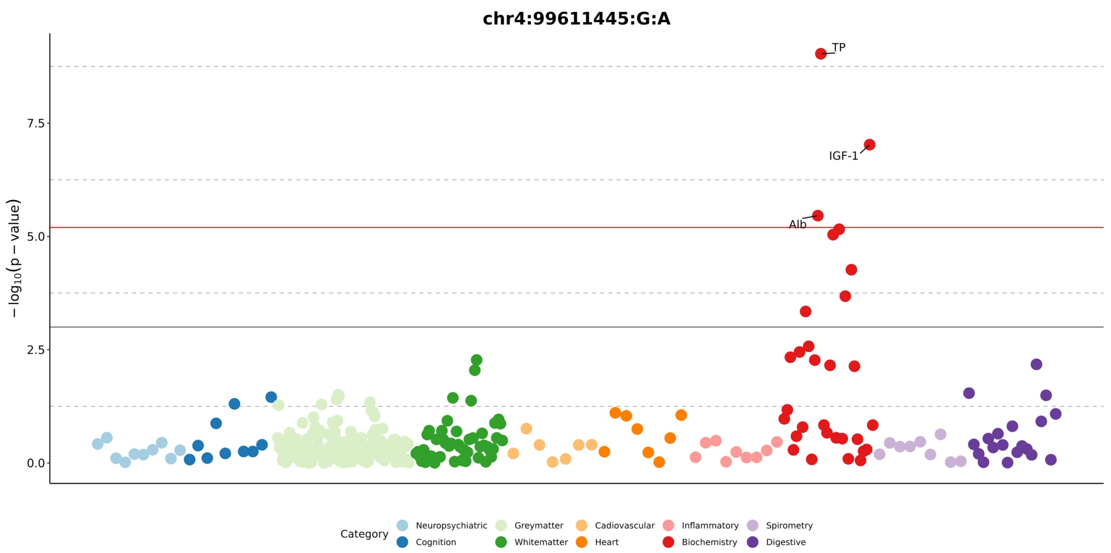

Supplementary Fig. 48 PheWAS results of variant chr4:99611445:G:A.

The x axis represented the phenotype categories and the y axis represented the -$\log_{10}(P)$. P-values shown are two-sided and unadjusted for multiple testing. The red horizontal line indicates the threshold for significant association (P <0.05/316/25=6.33×10^{-6}). The grey horizontal line denotes the threshold for a significant association to a lesser extent (P <0.001). Abbreviations: PheWAS, phenome-wide association studies; TP, Total Protein; IGF-1, IGF1; Alb, Albumin.

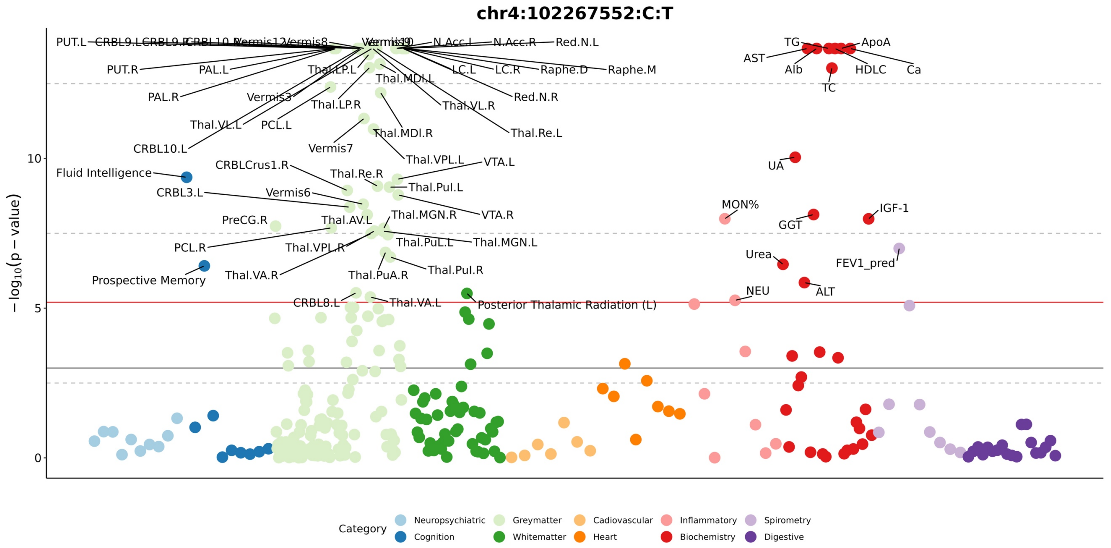

Supplementary Fig. 49 PheWAS results of variant chr4:102267552:C:T.

The *x* axis represented the phenotype categories and the *y* axis represented the $-\log_{10}(P)$. P-values shown are two-sided and unadjusted for multiple testing. The red horizontal line indicates the threshold for significant association ($P < 0.05/316/25 = 6.33\times10^{-6}$). The grey horizontal line denotes the threshold for a significant association to a lesser extent ($P < 0.001$). Values above $-\log_{10}(2.125\times10^{-14})$ are capped. Abbreviations: PheWAS, phenome-wide association studies; Vermis9, Vermis_9; HDLC, Hdl Cholesterol; PAL.R, Pallidum_R; Vermis10, Vermis_10; Raphe.M, Raphe_M; ApoA, Apolipoprotein A; Raphe.D, Raphe_D; PUT.L, Putamen_L; PAL.L, Pallidum_L; Vermis8, Vermis_8; Red.N.L, Red_N_L; Red.N.R, Red_N_R; LC.L, Lc_L; PUT.R, Putamen_R; AST, Aspartate Aminotransferase; LC.R, Lc_R; Thal.Re.L, Thal_Re_L; Ca, Calcium; CRBL9.R, Cerebellum_9_R; CRBL9.L, Cerebellum_9_L; Alb, Albumin; Vermis12, Vermis_1_2; Vermis3, Vermis_3; Thal.VL.R, Thal_Vl_R; Thal.VL.L, Thal_Vl_L; CRBL10.R, Cerebellum_10_R; N.Acc.L, N_Acc_L; TG, Triglycerides; N.Acc.R, N_Acc_R; CRBL10.L, Cerebellum_10_L; Thal.LP.L, Thal_Lp_L;

Thal.MDl.L, Thal_Mdl_L; Thal.LP.R, Thal_Lp_R; TC, Cholesterol; PCL.L, Paracentral_Lobule_L; Thal.MDl.R, Thal_Mdl_R; Vermis7, Vermis_7; Thal.VPL.L, Thal_Vpl_L; UA, Urate; Fluid Intelligence, Fluid Intelligence Score; VTA.L, Vta_L; Thal.Re.R, Thal_Re_R; Thal.PuI.L, Thal_Pui_L; CRBLCrus1.R, Cerebellum_Crus1_R; VTA.R, Vta_R; Vermis6, Vermis_6; CRBL3.L, Cerebellum_3_L; GGT, Gamma Glutamyltransferase; Thal.AV.L, Thal_Av_L; MON%, Monocyte Percentage; IGF-1, IGF1; PreCG.R, Precentral_R; PCL.R, Paracentral_Lobule_R; Thal.MGN.R, Thal_Mgn_R; Thal.VPL.R, Thal_Vpl_R; Thal.MGN.L, Thal_Mgn_L; Thal.VA.R, Thal_Va_R; Thal.PuL.L, Thal_Pul_L; FEV1_pred, Forced Expiratory Volume In 1-Second (Fev1), Predicted; Thal.PuA.R, Thal_Pua_R; Thal.PuI.R, Thal_Pui_R; Urea, Urea; Prospective Memory, Prospective Memory Result; ALT, Alanine Aminotransferase; CRBL8.L, Cerebellum_8_L; Posterior Thalamic Radiation (L), Posterior Thalamic Radiation (L); Thal.VA.L, Thal_Va_L; NEU, Neutrophill Count.

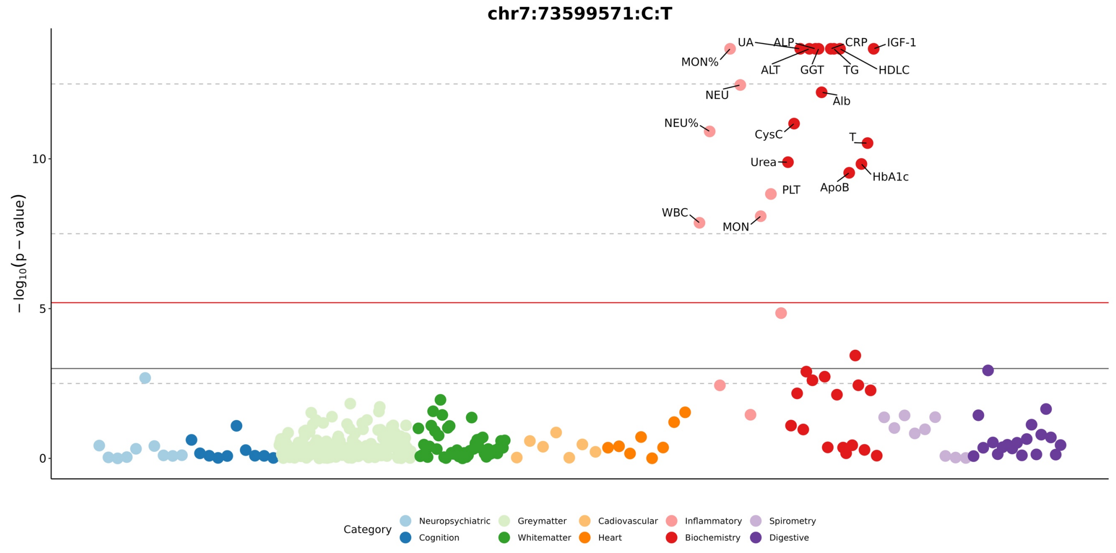

Supplementary Fig. 50 PheWAS results of variant chr7:73599571:C:T.

The *x* axis represented the phenotype categories and the *y* axis represented the -$\log_{10}(P)$. P-values shown are two-sided and unadjusted for multiple testing. The red horizontal line indicates the threshold for significant association (P <0.05/316/25=6.33×10^{-6}). The grey horizontal line denotes the threshold for a significant association to a lesser extent (P <0.001). Values above -$\log_{10}$ (2.125×10^{-14}) are capped. Abbreviations: PheWAS, phenome-wide association studies; TG, Triglycerides; GGT, Gamma Glutamyltransferase; UA, Urate; MON%, Monocyte Percentage; HDLC, Hdl Cholesterol; ALP, Alkaline Phosphatase; IGF-1, IGF1; CRP, C-Reactive Protein; ALT, Alanine Aminotransferase; NEU, Neutrophill Count; Alb, Albumin; CysC, Cystatin C; NEU%, Neutrophill Percentage; T, Testosterone; Urea, Urea; HbA1c, Glycated Haemoglobin (Hba1C); ApoB, Apolipoprotein B; PLT, Platelet Count; MON, Monocyte Count; WBC, White Blood Cell (Leukocyte) Count.

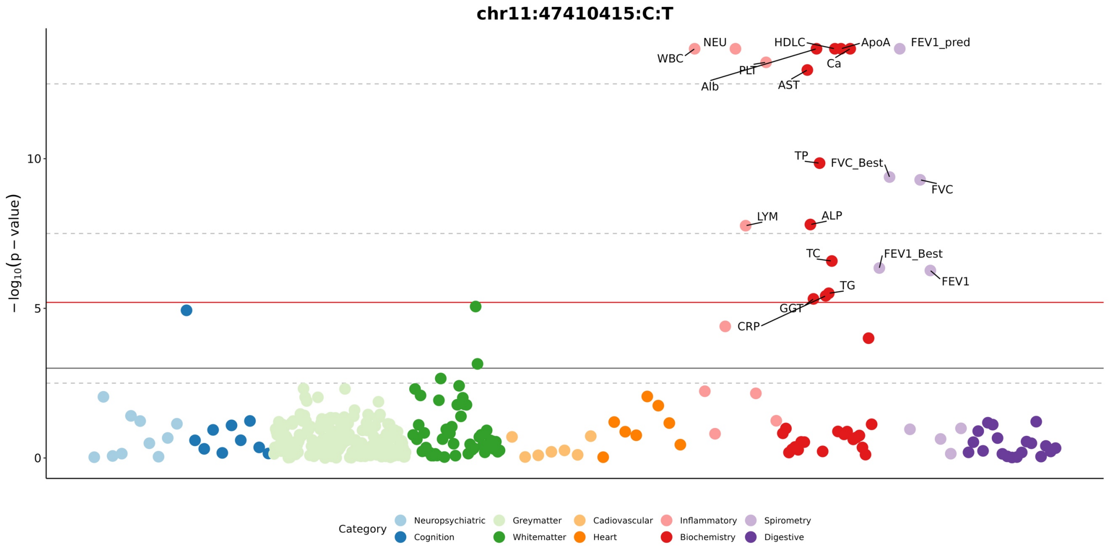

Supplementary Fig. 51 PheWAS results of variant chr11:47410415:C:T.

The x axis represented the phenotype categories and the y axis represented the $-\log_{10}(P)$. P-values shown are two-sided and unadjusted for multiple testing. The red horizontal line indicates the threshold for significant association ($P < 0.05/316/25 = 6.33\times10^{-6}$). The grey horizontal line denotes the threshold for a significant association to a lesser extent ($P < 0.001$). Values above $-\log_{10}(2.125\times10^{-14})$ are capped. Abbreviations: PheWAS, phenome-wide association studies; HDLC, Hdl Cholesterol; ApoA, Apolipoprotein A; Alb, Albumin; FEV1_pred, Forced Expiratory Volume In 1-Second (Fev1), Predicted; WBC, White Blood Cell (Leukocyte) Count; Ca, Calcium; NEU, Neutrophill Count; PLT, Platelet Count; AST, Aspartate Aminotransferase; TP, Total Protein; FVC_Best, Forced Vital Capacity (Fvc), Best Measure; FVC, Forced Vital Capacity (Fvc); ALP, Alkaline Phosphatase; LYM, Lymphocyte Count; TC, Cholesterol; FEV1_Best, Forced Expiratory Volume In 1-Second (Fev1), Best Measure; FEV1, Forced Expiratory Volume In 1-Second (Fev1); TG, Triglycerides; CRP, C-Reactive Protein; GGT, Gamma Glutamyltransferase.

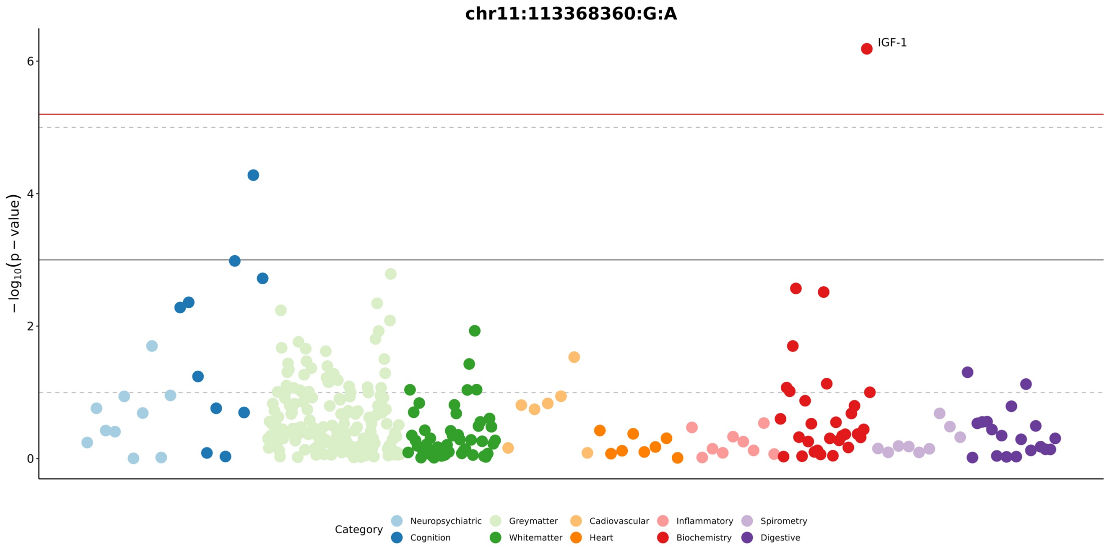

Supplementary Fig. 52 PheWAS results of variant chr11:113368360:G:A.

The *x* axis represented the phenotype categories and the *y* axis represented the $-\log_{10}(P)$. P-values shown are two-sided and unadjusted for multiple testing. The red horizontal line indicates the threshold for significant association (P $<0.05/316/25=6.33\times10^{-6}$). The grey horizontal line denotes the threshold for a significant association to a lesser extent (P <0.001). Abbreviations: PheWAS, phenome-wide association studies.

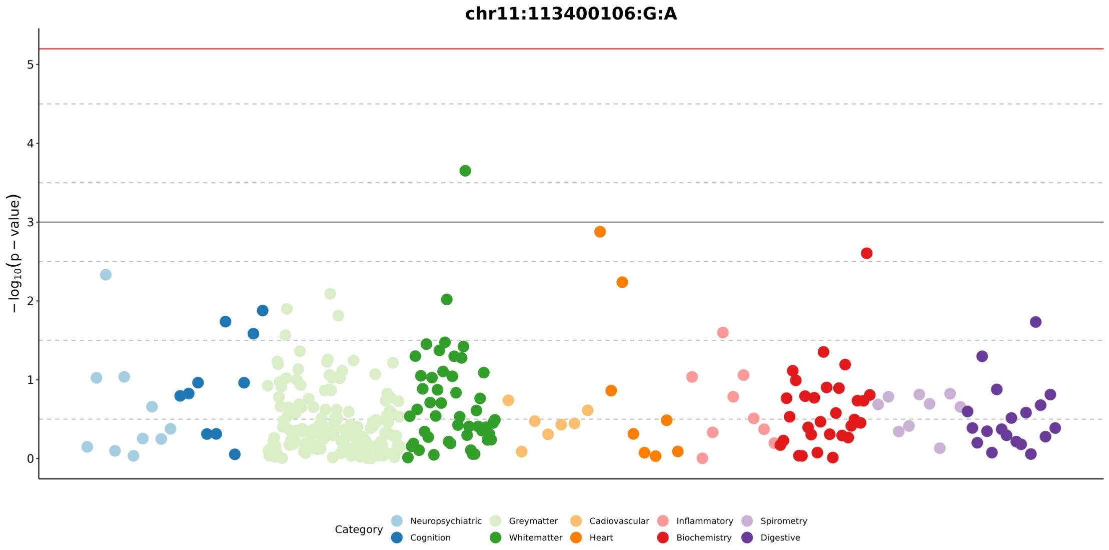

Supplementary Fig. 53 PheWAS results of variant chr11:113400106:G:A.

The *x* axis represented the phenotype categories and the *y* axis represented the -$\log_{10}(P)$. P-values shown are two-sided and unadjusted for multiple testing. The red horizontal line indicates the threshold for significant association (P <0.05/316/25=6.33×10^{-6}). The grey horizontal line denotes the threshold for a significant association to a lesser extent (P <0.001). Abbreviations: PheWAS, phenome-wide association studies.

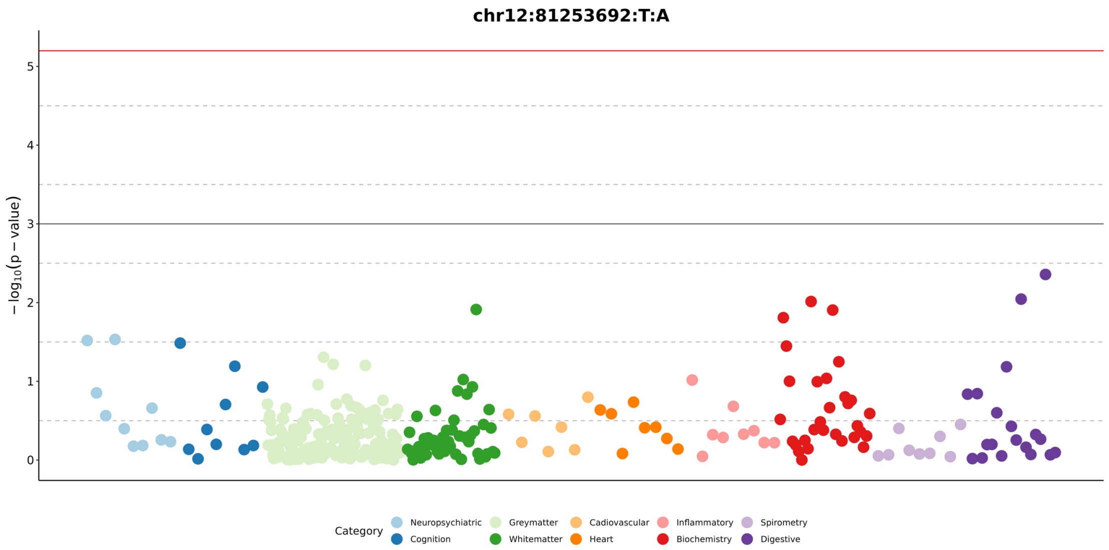

Supplementary Fig. 54 PheWAS results of variant chr12:81253692:T:A.

The *x* axis represented the phenotype categories and the *y* axis represented the $-\log_{10}(P)$. P-values shown are two-sided and unadjusted for multiple testing. The red horizontal line indicates the threshold for significant association ($P < 0.05/316/25 = 6.33\times10^{-6}$). The grey horizontal line denotes the threshold for a significant association to a lesser extent ($P < 0.001$). Abbreviations: PheWAS, phenome-wide association studies.

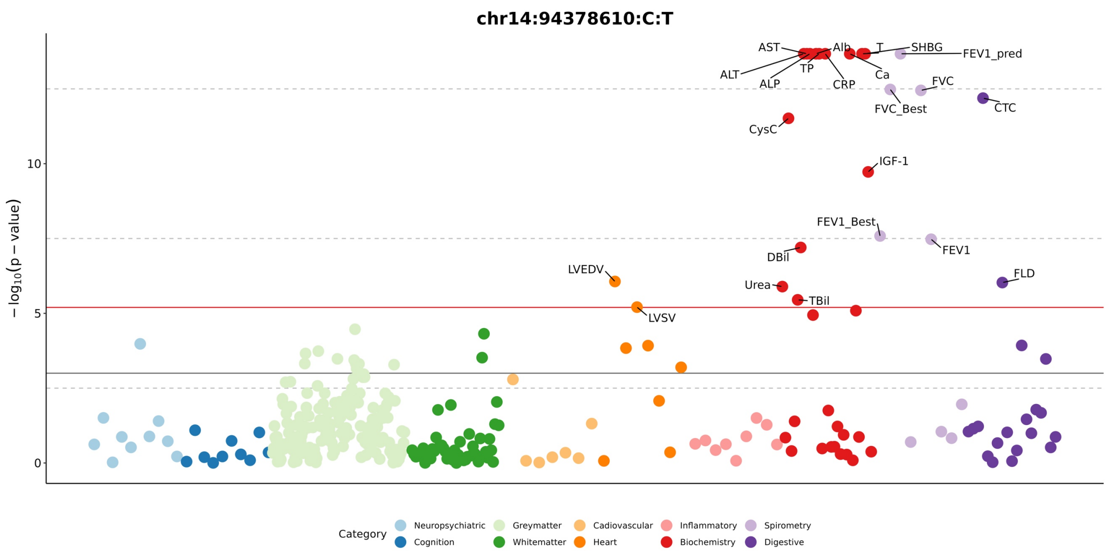

Supplementary Fig. 55 PheWAS results of variant chr14:94378610:C:T.

The *x* axis represented the phenotype categories and the *y* axis represented the $-\log_{10}(P)$. P-values shown are two-sided and unadjusted for multiple testing. The red horizontal line indicates the threshold for significant association ($P < 0.05/316/25 = 6.33\times10^{-6}$). The grey horizontal line denotes the threshold for a significant association to a lesser extent ($P < 0.001$). Values above $-\log_{10}(2.125\times10^{-14})$ are capped. Abbreviations: PheWAS, phenome-wide association studies; Alb, Albumin; SHBG, Sex Hormone-Binding Globulin, Shbg; TP, Total Protein; ALT, Alanine Aminotransferase; ALP, Alkaline Phosphatase; FEV1_pred, Forced Expiratory Volume In 1-Second (Fev1), Predicted; Ca, Calcium; AST, Aspartate Aminotransferase; CRP, C-Reactive Protein; T, Testosterone; FVC_Best, Forced Vital Capacity (Fvc), Best Measure; FVC, Forced Vital Capacity (Fvc); CTC, Cholelithiasis; CysC, Cystatin C; IGF-1, IGF1; FEV1_Best, Forced Expiratory Volume In 1-Second (Fev1), Best Measure; FEV1, Forced Expiratory Volume In 1-Second (Fev1); DBil, Direct Bilirubin; LVEDV, Lv End Diastolic Volume; FLD, Fibrosis Liver Disease; Urea, Urea; TBil, Total Bilirubin; LVSV, Lv

Stroke Volume.

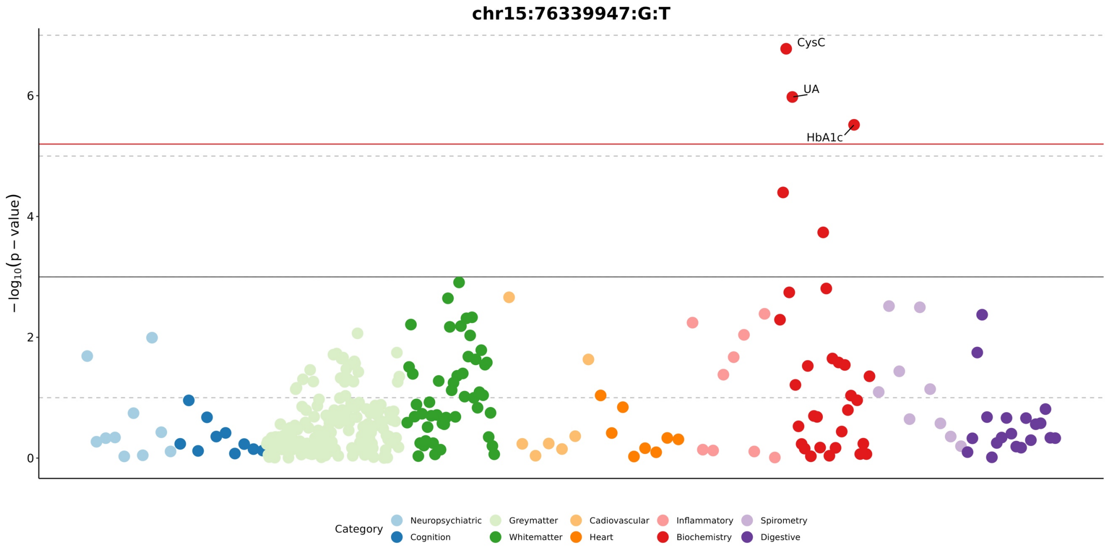

Supplementary Fig. 56 PheWAS results of variant chr15:76339947:G:T.

The *x* axis represented the phenotype categories and the *y* axis represented the $-\log_{10}(P)$. P-values shown are two-sided and unadjusted for multiple testing. The red horizontal line indicates the threshold for significant association (P <0.05/316/25=6.33×10^{-6}). The grey horizontal line denotes the threshold for a significant association to a lesser extent (P <0.001). Abbreviations: PheWAS, phenome-wide association studies; Cr, Creatinine; CysC, Cystatin C; HbA1c, Glycated Haemoglobin.

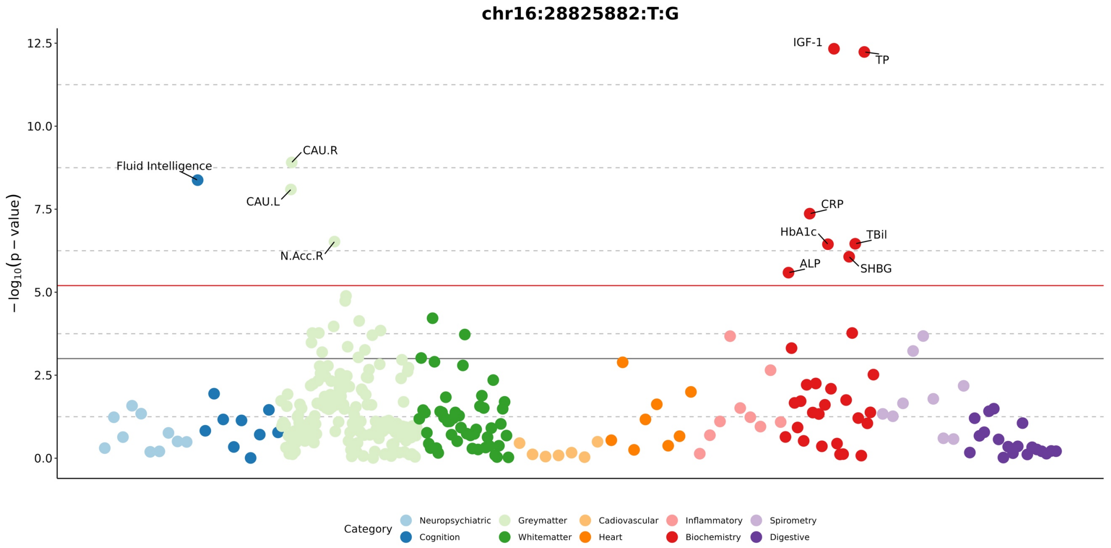

Supplementary Fig. 57 PheWAS results of variant chr16:28825882:T:G.

The *x* axis represented the phenotype categories and the *y* axis represented the $-\log_{10}(P)$. P-values shown are two-sided and unadjusted for multiple testing. The red horizontal line indicates the threshold for significant association (P <0.05/316/25=6.33×10^{-6}). The grey horizontal line denotes the threshold for a significant association to a lesser extent (P <0.001). Abbreviations: PheWAS, phenome-wide association studies; IGF-1, IGF1; TP, Total Protein; CAU.R, Caudate_R; Fluid Intelligence, Fluid Intelligence Score; CAU.L, Caudate_L; CRP, C-Reactive Protein; N.Acc.R, N_Acc_R; TBil, Total Bilirubin; HbA1c, Glycated Haemoglobin (Hba1C); SHBG, Sex Hormone-Binding Globulin, Shbg; ALP, Alkaline Phosphatase.

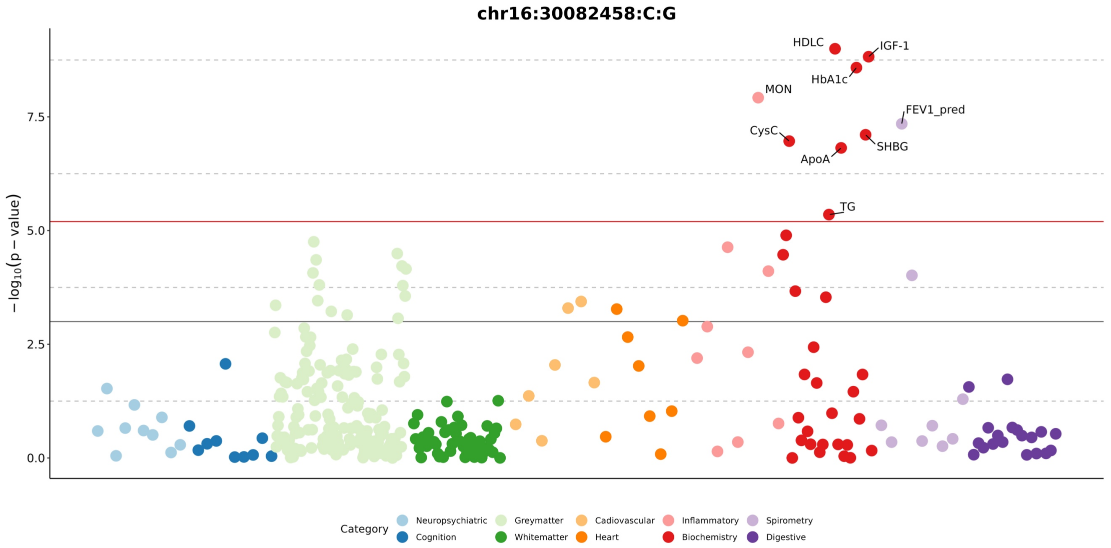

Supplementary Fig. 58 PheWAS results of variant chr16:30082458:C:G.

The x axis represented the phenotype categories and the y axis represented the -log$_{10}$(P). P-values shown are two-sided and unadjusted for multiple testing. The red horizontal line indicates the threshold for significant association (P <0.05/316/25=6.33×10^{-6}). The grey horizontal line denotes the threshold for a significant association to a lesser extent (P <0.001). Values above -log$_{10}$ (2.125×10^{-14}) are capped. Abbreviations: PheWAS, phenome-wide association studies; HDLC, Hdl Cholesterol; IGF-1, IGF1; HbA1c, Glycated Haemoglobin (Hba1C); MON, Monocyte Count; FEV1_pred, Forced Expiratory Volume In 1-Second (Fev1), Predicted; SHBG, Sex Hormone-Binding Globulin, Shbg; CysC, Cystatin C; ApoA, Apolipoprotein A; TG, Triglycerides.

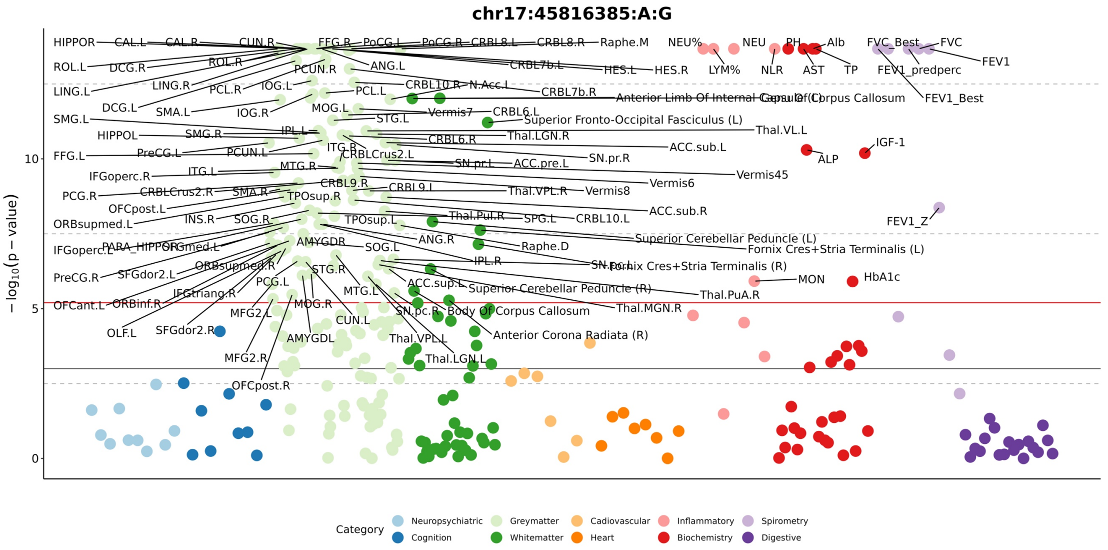

Supplementary Fig. 59 PheWAS results of variant chr17:45816385:A:G.

The x axis represented the phenotype categories and the y axis represented the $-\log_{10}(P)$. P-values shown are two-sided and unadjusted for multiple testing. The red horizontal line indicates the threshold for significant association ($P < 0.05/316/25 = 6.33\times10^{-6}$). The grey horizontal line denotes the threshold for a significant association to a lesser extent ($P < 0.001$). Values above $-\log_{10}(2.125\times10^{-14})$ are capped. Abbreviations: PheWAS, phenome-wide association studies; FVC, Forced Vital Capacity (Fvc); FVC_Best, Forced Vital Capacity (Fvc), Best Measure; FEV1_Best, Forced Expiratory Volume In 1-Second (Fev1), Best Measure; FEV1, Forced Expiratory Volume In 1-Second (Fev1); FEV1_predperc, Forced Expiratory Volume In 1-Second (Fev1), Predicted Percentage; NEU%, Neutrophill Percentage; LING.R, Lingual_R; CAL.R, Calcarine_R; NLR, Neutrophill Lymphocyte Ratio; TP, Total Protein; LING.L, Lingual_L; HES.L, Heschl_L; LYM%, Lymphocyte Percentage; CRBL8.R, Cerebellum_8_R; HIPPOR, Hippocampus_R; ROL.L, Rolandic_Oper_L; AST, Aspartate Aminotransferase; FFG.R, Fusiform_R; Raphe.M, Raphe_M; PH, Phosphate; ANG.L,

Angular_L; Alb, Albumin; NEU, Neutrophill Count; CAL.L, Calcarine_L; CRBL8.L, Cerebellum_8_L; CUN.R, Cuneus_R; HES.R, Heschl_R; PoCG.L, Postcentral_L; DCG.R, Cingulate_Mid_R; PoCG.R, Postcentral_R; CRBL7b.L, Cerebellum_7B_L; ROL.R, Rolandic_Oper_R; PCL.R, Paracentral_Lobule_R; DCG.L, Cingulate_Mid_L; PCUN.R, Precuneus_R; CRBL7b.R, Cerebellum_7B_R; IOG.L, Occipital_Inf_L; CRBL10.R, Cerebellum_10_R; PCL.L, Paracentral_Lobule_L; IOG.R, Occipital_Inf_R; MOG.L, Occipital_Mid_L; Anterior Limb Of Internal Capsule (L), Anterior Limb Of Internal Capsule (L); Genu Of Corpus Callosum, Genu Of Corpus Callosum; N.Acc.L, N_Acc_L; SMA.L, Supp_Motor_Area_L; Vermis7, Vermis_7; CRBL6.L, Cerebellum_6_L; STG.L, Temporal_Sup_L; Superior Fronto-Occipital Fasciculus (L), Superior Fronto-Occipital Fasciculus (L); IPL.L, Parietal_Inf_L; Thal.VL.L, Thal_Vl_L; SMG.L, Supramarginal_L; SMG.R, Supramarginal_R; Thal.LGN.R, Thal_Lgn_R; CRBLCrus2.L, Cerebellum_Crus2_L; CRBL6.R, Cerebellum_6_R; HIPPOL, Hippocampus_L; PCUN.L, Precuneus_L; ACC.sub.L, Acc_Sub_L; PreCG.L, Precentral_L; SN.pr.R, Sn_Pr_R; ALP, Alkaline Phosphatase; IGF-1, IGF1; FFG.L, Fusiform_L; ACC.pre.L, Acc_Pre_L; SN.pr.L, Sn_Pr_L; ITG.R, Temporal_Inf_R; Vermis45, Vermis_4_5; MTG.R, Temporal_Mid_R; ITG.L, Temporal_Inf_L; Vermis6, Vermis_6; IFGoperc.R, Frontal_Inf_Oper_R; Vermis8, Vermis_8; CRBL9.R, Cerebellum_9_R; CRBLCrus2.R, Cerebellum_Crus2_R; PCG.R, Cingulate_Post_R; SMA.R, Supp_Motor_Area_R; OFCpost.L, Ofcpost_L; CRBL9.L, Cerebellum_9_L; Thal.VPL.R, Thal_Vpl_R; ORBsupmed.L, Frontal_Med_Orb_L; ACC.sub.R, Acc_Sub_R; CRBL10.L, Cerebellum_10_L; TPOsup.R, Temporal_Pole_Sup_R; INS.R, Insula_R; FEV1_Z, Forced

Expiratory Volume In 1-Second (Fev1) Z-Score; SOG.R, Occipital_Sup_R; Thal.PuI.R, Thal_Pui_R; SPG.L, Parietal_Sup_L; TPOsup.L, Temporal_Pole_Sup_L; PARA_HIPPOR, Parahippocampal_R; Superior Cerebellar Peduncle (L), Superior Cerebellar Peduncle (L); IFGoperc.L, Frontal_Inf_Oper_L; IPL.R, Parietal_Inf_R; ANG.R, Angular_R; Raphe.D, Raphe_D; SFGmed.L, Frontal_Sup_Medial_L; Fornix Cres+Stria Terminalis (L), Fornix Cres+Stria Terminalis (L); PreCG.R, Precentral_R; AMYGDR, Amygdala_R; OFCant.L, Ofcant_L; SOG.L, Occipital_Sup_L; SFGdor2.L, Frontal_Sup_2_L; Fornix Cres+Stria Terminalis (R), Fornix Cres+Stria Terminalis (R); ORBinf.R, Frontal_Inf_Orb_2_R; ORBsupmed.R, Frontal_Med_Orb_R; OLF.L, Olfactory_L; IFGtriang.R, Frontal_Inf_Tri_R; MTG.L, Temporal_Mid_L; SFGdor2.R, Frontal_Sup_2_R; SN.pc.L, Sn_Pc_L; Thal.PuA.R, Thal_Pua_R; PCG.L, Cingulate_Post_L; CUN.L, Cuneus_L; Thal.MGN.R, Thal_Mgn_R; STG.R, Temporal_Sup_R; ACC.sup.L, Acc_Sup_L; Superior Cerebellar Peduncle (R), Superior Cerebellar Peduncle (R); MOG.R, Occipital_Mid_R; AMYGDL, Amygdala_L; Thal.VPL.L, Thal_Vpl_L; MON, Monocyte Count; HbA1c, Glycated Haemoglobin (Hba1C); MFG2.L, Frontal_Mid_2_L; Thal.LGN.L, Thal_Lgn_L; Body Of Corpus Callosum, Body Of Corpus Callosum; SN.pc.R, Sn_Pc_R; OFCpost.R, Ofcpost_R; MFG2.R, Frontal_Mid_2_R; Anterior Corona Radiata (R), Anterior Corona Radiata (R).

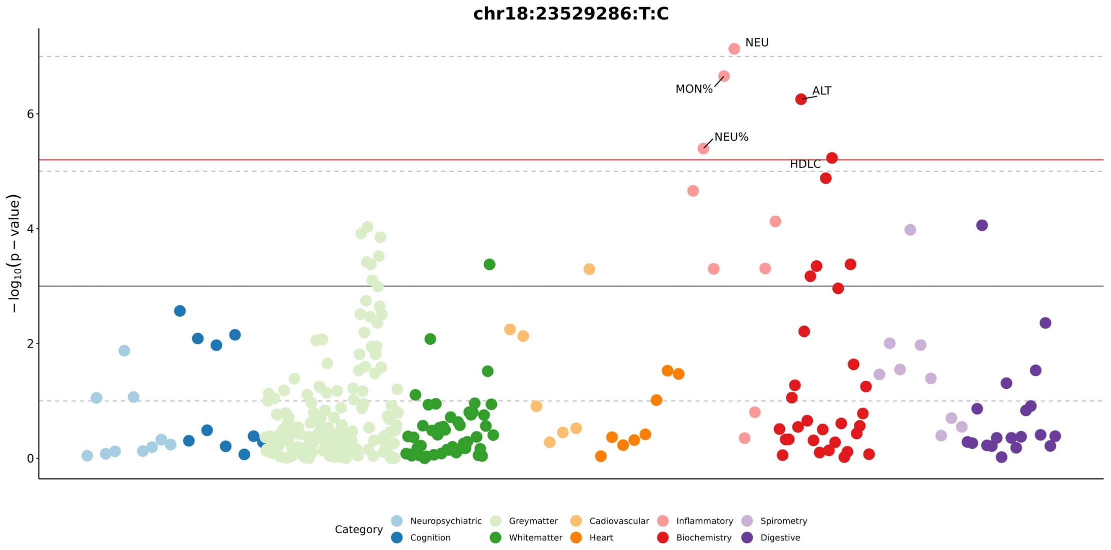

Supplementary Fig. 60 PheWAS results of variant chr18:23529286:T:C.

The *x* axis represented the phenotype categories and the *y* axis represented the -$\log_{10}(P)$. P-values shown are two-sided and unadjusted for multiple testing. The red horizontal line indicates the threshold for significant association (P <0.05/316/25=6.33×10^{-6}). The grey horizontal line denotes the threshold for a significant association to a lesser extent (P <0.001). Abbreviations: PheWAS, phenome-wide association studies; NEU%, Neutrophill Percentage; MON%, Monocyte Percentage; NEU, Neutrophill Count; ALT, Alanine Aminotransferase; TG, Triglycerides; HDLC, Hdl Cholesterol.

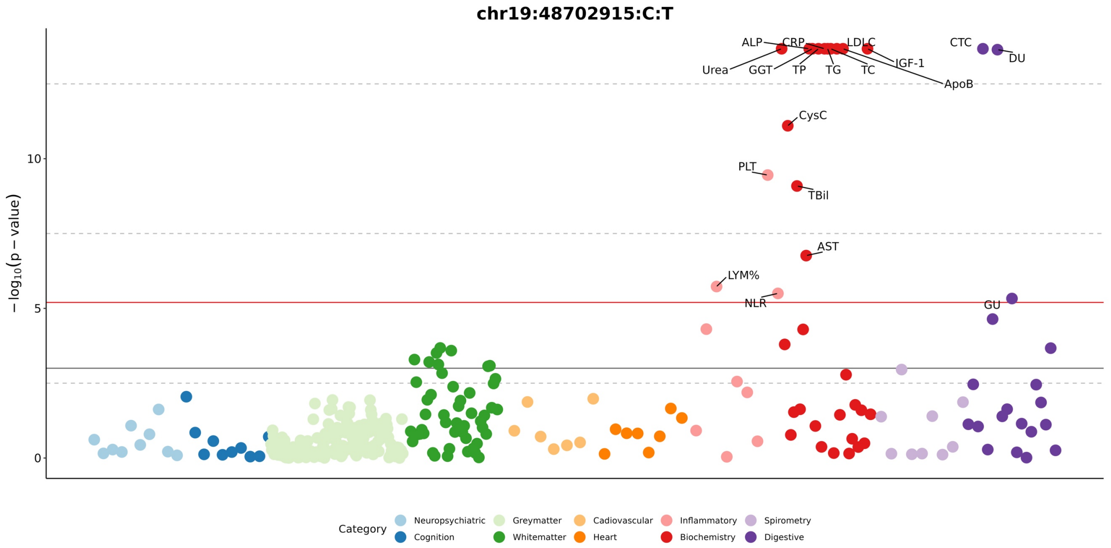

Supplementary Fig. 61 PheWAS results of variant chr19:48702915:C:T.

The x axis represented the phenotype categories and the y axis represented the $-log_{10}(P)$. P-values shown are two-sided and unadjusted for multiple testing. The red horizontal line indicates the threshold for significant association ($P < 0.05/316/25 = 6.33\times10^{-6}$). The grey horizontal line denotes the threshold for a significant association to a lesser extent ($P < 0.001$). Values above $-\log_{10}(2.125\times10^{-14})$ are capped. Abbreviations: PheWAS, phenome-wide association studies; ALP, Alkaline Phosphatase; GGT, Gamma Glutamyltransferase; Urea, Urea; TP, Total Protein; ApoB, Apolipoprotein B; TC, Cholesterol; LDLC, Ldl Direct; IGF-1, IGF1; CRP, C-Reactive Protein; CTC, Cholelithiasis; TG, Triglycerides; DU, Duodenal Ulcer Disease; CysC, Cystatin C; PLT, Platelet Count; TBil, Total Bilirubin; AST, Aspartate Aminotransferase; LYM%, Lymphocyte Percentage; NLR, Neutrophill Lymphocyte Ratio; GU, Gastric Ulcer Disease.
