# Supplementary material for: Whole exome sequencing analysis identifies genes for alcohol consumption
Source: Nat Commun. 2024 Jul 10;15:5777. doi: 10.1038/s41467-024-50132-3 (PMC11233704; doi:10.1038/s41467-024-50132-3)
Supplement: Supplementary file 3 — Description of Additional Supplementary Files [file 41467_2024_50132_MOESM3_ESM.pdf]

File Name: Supplementary Data 1
Description: Description of phenotype used in the main analyses. Detailed information for each phenotype is available at http://biobank.ctsu.ox.ac.uk/showcase/search.cgi.

File Name: Supplementary Data 2
Description: Demographics of the study sample. Abbreviations: SD, standard deviation; Pctile, Percentile.

File Name: Supplementary Data 3
Description: Exome-wide significant variants for alcohol consumption. SAIGE GENE+ was used to perform single-variant association tests (two-sided). No adjustments were made for multiple comparisons. Details are available at https://saigegit.github.io/SAIGE-doc/docs/single_step2.html. Abbreviations: CHR, chromosome; POS, position; AF_Allele2, allele 2 frequency in the analyzed sample; BETA, Beta coefficient for allele 2; SE, standard error; Tstat, t statistic for allele 2.

File Name: Supplementary Data 4
Description: Replication results of the identified single variants using a large GWAS study in participants excluding participants from UK Biobank. Data were from public GWAS summary statistics. No adjustments were made for multiple comparisons. Abbreviations: CHROM, chromosome; POS, position; REF, reference allele; ALT, alternative allele; AF, allele frequency; BETA, Beta coefficient for alternative allele; SE, Standard error of the beta; STAT, Chi-square statistic; N, Sum of sample size across contributing cohorts; Effective_N, Sum of Sample size * imputation r2 across contributing cohorts.

File Name: Supplementary Data 5
Description: Association analysis between the identified single variants and alcohol use disorder using FinnGen study. Data were from public GWAS summary statistics. No adjustments were made for multiple comparisons. Abbreviations: CHR, chromosome; POS, position; REF, reference allele; ALT, alternative allele (effect allele); maf, minor allele frequency; beta, effect size (log(OR) scale) estimated with regenie for the alternative allele; sebeta, standard error of effect size estimated with regenie. See https://finngen.gitbook.io/documentation/v/r9/data-description for details.

File Name: Supplementary Data 6
Description: ExWAS for single variants after adjusting rs1229984. SAIGE GENE+ was used to perform single-variant association tests (two-sided). No adjustments were made for multiple comparisons. Abbreviations: CHR, chromosome; POS, position; AF_Allele2, allele 2 frequency in the analyzed sample; BETA, Beta coefficient for Allele 2; SE, standard error; Tstat, t statistic for Allele 2.

File Name: Supplementary Data 7
Description: ExWAS for single variants after exclusion of former drinkers and non-

drinkers. SAIGE GENE+ was used to perform single-variant association tests (two-sided). No adjustments were made for multiple comparisons. Abbreviations: CHR, chromosome; POS, position; AF_Allele2, allele 2 frequency in the analyzed sample; BETA, Beta coefficient for Allele 2; SE, standard error; Tstat, t statistic for Allele 2.

File Name: Supplementary Data 8
Description: Exome-wide significant variants for AUDIT. SAIGE GENE+ was used to perform single-variant association tests (two-sided). No adjustments were made for multiple comparisons. Abbreviations: CHR, chromosome; POS, position; AF_Allele2, frequency of allele 2 in the analyzed sample; BETA, Beta coefficient for Allele 2; SE, standard error; Tstat, t statistic for Allele 2.

File Name: Supplementary Data 9
Description: Significant associations of alcohol consumption at FDR Q < 0.05. SAIGE GENE+ was used to perform gene-based association tests (two-sided). No adjustments were made for multiple comparisons. Details are available at https://saigegit.github.io/SAIGE-doc/docs/set_step2.html.

File Name: Supplementary Data 10
Description: Burden heritability estimates for alcohol consumption. Burden Heritability Regression (BHR) was used to estimate the heritability explained by mutational burden in each gene-set. total_h2: total heritability.

File Name: Supplementary Data 11
Description: Gene-based collapsing association analysis after exclusion of former drinkers and non-drinkers. SAIGE GENE+ was used to perform gene-based association tests. No adjustments were made for multiple comparisons.

File Name: Supplementary Data 12
Description: Gene-based collapsing association analysis after adjusting rs1229984. SAIGE GENE+ was used to perform gene-based association tests. No adjustments were made for multiple comparisons.

File Name: Supplementary Data 13
Description: Gene-based collapsing association analysis of AUDIT. SAIGE GENE+ was used to perform gene-based association tests. No adjustments were made for multiple comparisons.

File Name: Supplementary Data 14
Description: Results of leave-one-variant-out analysis. SAIGE GENE+ was used to perform gene-based association tests. No adjustments were made for multiple comparisons.

File Name: Supplementary Data 15

Description: Results of the conditional analysis. SAIGE GENE+ was used to perform gene-based association tests. No adjustments were made for multiple comparisons.

File Name: Supplementary Data 16
Description: Sex-specific associations between significant genes and alcohol consumption. SAIGE GENE+ was used to perform gene-based association tests. No adjustments were made for multiple comparisons.

File Name: Supplementary Data 17
Description: Associations of rare variants in previously reported GWAS genes. SAIGE GENE+ was used to perform gene-based association tests. No adjustments were made for multiple comparisons.

File Name: Supplementary Data 18
Description: GO enrichment analysis of alcohol consumption genes. The gProfiler software was used to perform enrichment analysis. The default option g:SCS method was used for multiple testing correction.

File Name: Supplementary Data 19
Description: Tissue-specificity analysis with gene expression patterns from the Human Protein Atlas project. The R package TissueEnrich to perform the enrichment analysis. TissueEnrich used a hypergeometric test to calculate the enrichment of the tissue-specific genes using RNA sequencing data from the Human Protein Atlas.

File Name: Supplementary Data 20
Description: Similarities between ANKRD12 and the top 10 closest genes. Details are available at https://astrazeneca-cgr-publications.github.io/gene-scout/genescout.html?trait_radio_option=ag_all_traits_without_telomere&query_gene_input=ANKRD12.

File Name: Supplementary Data 21
Description: Enrichment analysis of the top 10 similar genes plus ANKRD12. Enrichment analyses were performed using Gene-SCOUT. Fisher's exact test was performed. Details are available at https://astrazeneca-cgr-publications.github.io/gene-scout/genescout.html?trait_radio_option=ag_all_traits_without_telomere&query_gene_input=ANKRD12.

File Name: Supplementary Data 22
Description: Phenotypes used in the PheWAS analysis. Detailed information for each phenotype is available at http://biobank.ctsu.ox.ac.uk/showcase/search.cgi.

File Name: Supplementary Data 23
Description: Phenotypic associations of genes identified by burden test. SAIGE

GENE+ was used to perform gene-based association tests (two-sided). No adjustments were made for multiple comparisons.

File Name: Supplementary Data 24
Description: Phenotypic associations of variants identified by single-variant analysis. SAIGE GENE+ was used to perform single-variant association tests (two-sided). No adjustments were made for multiple comparisons.

File Name: Supplementary Data 25
Description: Mendelian randomization analysis between cognition and alcohol consumption. The R package TwoSampleMR was used to perform Mendelian randomization analysis. P values are two-sided. No adjustments were made for multiple comparisons. Abbreviations: nsnp, number of SNPs; b, beta value; se, standard error of beta; pval, p value; low_ci, lower bound of the 95% confidence interval; up_ci, upper bound of the 95% confidence interval.

File Name: Supplementary Data 26
Description: ExWAS for single variants using all white British participants. SAIGE GENE+ was used to perform single-variant association tests (two-sided). No adjustments were made for multiple comparisons. Abbreviations: CHR, chromosome; POS, position; AF_Allele2, allele 2 frequency in the analyzed sample; BETA, Beta coefficient for Allele 2; SE, standard error; Tstat, t statistic for Allele 2, Overlapped: whether the variant is overlapped in the primary analysis.

File Name: Supplementary Data 27
Description: Gene-based collapsing association analysis using all white British participants. SAIGE GENE+ was used to perform gene-based association tests. No adjustments were made for multiple comparisons.

File Name: Supplementary Data 28
Description: ExWAS for single variants in unrelated non-white participants. SAIGE GENE+ was used to perform single-variant association tests (two-sided). No adjustments were made for multiple comparisons.
