# Supplementary material for: Whole exome sequencing analysis identifies genes for alcohol consumption
Source: Nat Commun. 2024 Jul 10;15:5777. doi: 10.1038/s41467-024-50132-3 (PMC11233704; doi:10.1038/s41467-024-50132-3)
Supplement: Supplementary file 5 — Reporting Summary [file 41467_2024_50132_MOESM5_ESM.pdf]

nature portfolio

Corresponding author(s): Jianfeng Feng, Wei Cheng, Jin-Tai Yu

Last updated by author(s): Apr 21, 2024

Reporting Summary

Nature Portfolio wishes to improve the reproducibility of the work that we publish. This form provides structure for consistency and transparency in reporting. For further information on Nature Portfolio policies, see our Editorial Policies and the Editorial Policy Checklist.

Statistics

For all statistical analyses, confirm that the following items are present in the figure legend, table legend, main text, or Methods section.

n/a	Confirmed	
☐	☒	The exact sample size (*n*) for each experimental group/condition, given as a discrete number and unit of measurement
☐	☒	A statement on whether measurements were taken from distinct samples or whether the same sample was measured repeatedly
☐	☒	The statistical test(s) used AND whether they are one- or two-sided<br>*Only common tests should be described solely by name; describe more complex techniques in the Methods section.*
☐	☒	A description of all covariates tested
☐	☒	A description of any assumptions or corrections, such as tests of normality and adjustment for multiple comparisons
☐	☒	A full description of the statistical parameters including central tendency (e.g. means) or other basic estimates (e.g. regression coefficient) AND variation (e.g. standard deviation) or associated estimates of uncertainty (e.g. confidence intervals)
☐	☒	For null hypothesis testing, the test statistic (e.g. *F*, *t*, *r*) with confidence intervals, effect sizes, degrees of freedom and *P* value noted<br>*Give P values as exact values whenever suitable.*
☒	☐	For Bayesian analysis, information on the choice of priors and Markov chain Monte Carlo settings
☒	☐	For hierarchical and complex designs, identification of the appropriate level for tests and full reporting of outcomes
☒	☐	Estimates of effect sizes (e.g. Cohen's *d*, Pearson's *r*), indicating how they were calculated

Our web collection on statistics for biologists contains articles on many of the points above.

Software and code

Policy information about availability of computer code

Data collection	No software used during data collection, and data can be directly download after approval of access application. The UK Biobank data collection can be found at https://www.ukbiobank.ac.uk/enable-your-research.
Data analysis	The code used for the ExWAS analyses and PheWas analyses was an adaptation of the R package SAIGE GENE+ and was available through the GitHub repository: https://github.com/saigegit/SAIGE. Burden heritability regression analysis was performed via the R package BHR (v.0.1.0), which source code was publicly available at the GitHub repository: https://github.com/ajaynadig/bhr. Annotation of significant variants was performed using VEP (https://asia.ensembl.org/Homo_sapiens/Tools/VEP). Gene ontology analysis was performed using g:Profiler (https://biit.cs.ut.ee/gprofiler/gost) and tissue enrichment analysis was performed via the R package TissueEnrich (v.1.16.0, https://github.com/Tuteja-Lab/TissueEnrich). The code for the main analysis and visualization of single-cell RNA sequencing data was an adaptation of the R package Seurat (v.4.3.0) and was available through the website: https://satijalab.org/seurat/index.html. Gene-Scout was available through the website: https://astrazeneca-cgr-publications.github.io/gene-scout/.

For manuscripts utilizing custom algorithms or software that are central to the research but not yet described in published literature, software must be made available to editors and reviewers. We strongly encourage code deposition in a community repository (e.g. GitHub). See the Nature Portfolio guidelines for submitting code & software for further information.

Data

Policy information about availability of data

All manuscripts must include a data availability statement. This statement should provide the following information, where applicable:

- Accession codes, unique identifiers, or web links for publicly available datasets
- A description of any restrictions on data availability
- For clinical datasets or third party data, please ensure that the statement adheres to our policy

This research has been conducted using data from UK Biobank under Application Numbers 19542. The individual-level phenotypic and genetic data are available under restricted access, access can be obtained by application through the UK Biobank platform (https://www.ukbiobank.ac.uk/). The scRNA-seq data used in this study are available in the GEO database under accession code GSE115469 (https://www.ncbi.nlm.nih.gov/geo/query/acc.cgi?acc=GSE115469) and GSE173731 (https://www.ncbi.nlm.nih.gov/geo/query/acc.cgi?acc=GSE173731). The transcript expression data used in this study are available in the Human Protein Atlas database (https://v22.proteinatlas.org/about/download). The processed human mRNA-seq data used in the present study were available in the PsychENCODE study (http://development.psychencode.org/). Summary statistics from FinnGen are available at https://storage.googleapis.com/finngen-public-data-r9/summary_stats/finngen_R9_AUD.gz and https://storage.googleapis.com/finngen-public-data-r9/summary_stats/finngen_R9_AUD_SWEDISH.gz. KEGG database used in gProfiler are available at https://www.genome.jp/kegg/. The Gene Ontology database used in gProfiler are available at https://www.ensembl.org/.

Research involving human participants, their data, or biological material

Policy information about studies with human participants or human data. See also policy information about sex, gender (identity/presentation), and sexual orientation and race, ethnicity and racism.

Reporting on sex and gender	We took sex into considerations in our study and our findings could apply to both male and female. Sex (Field ID 31, https://biobank.ctsu.ox.ac.uk/showcase/field.cgi?id=31) in the UK Biobank was determined based on self-reporting data via questionaire. Participants with discordant self-reported sex and genetic sex were excluded from our analyses. All included participants gave written informed consent for sharing of individual-level data.
Reporting on race, ethnicity, or other socially relevant groupings	Ethnic background (UKB Field ID 21000) was used to include the white British participants in the main analysis. Samples who self-reported 'White British' and have very similar genetic ancestry based on a principal components analysis of the genotypes were defined as the white British participants and used in the main analysis. See https://biobank.ndph.ox.ac.uk/ukb/ukb/docs/ukb_genetic_data_description.txt for details.
Population characteristics	In the main analysis, 304,119 individuals (164,494 [54.09%] female; mean age at baseline, 56.87 years) were included. The baseline demographic data of participants is shown in Supplementary Table 2. Sex-disaggregated demographic data is shown in Supplementary Table 2.
Recruitment	The UKB enrolled the participants aged 37-73 years between 2006 and 2010 for baseline assessments in 22 centers across the UK. The assessment visits comprised interviews and questionnaires covering lifestyles and health conditions, physical measures, biological samples, imaging, and genotyping. The database is linked to national health datasets, including primary care, hospital inpatient, death, and cancer registration data.
Ethics oversight	UK Biobank has received ethical approval from the North West Multi-centre Research Ethics Committee (MREC, https://www.ukbiobank.ac.uk/learn-more-about-uk-biobank/about-us/ethics), and informed consent through electronic signature was obtained from study participants. This study utilized the UK Biobank Resource under application number 19542.

Note that full information on the approval of the study protocol must also be provided in the manuscript.

Field-specific reporting

Please select the one below that is the best fit for your research. If you are not sure, read the appropriate sections before making your selection.

[x] Life sciences [ ] Behavioural & social sciences [ ] Ecological, evolutionary & environmental sciences

For a reference copy of the document with all sections, see nature.com/documents/nr-reporting-summary-flat.pdf

Life sciences study design

All studies must disclose on these points even when the disclosure is negative.

Sample size	No statistical methods were used to predetermine sample sizes. All currently available sample in the UK Biobank were included.
Data exclusions	Participants without whole-exome sequencing data, and those failed to pass quality control were excluded. Details of data quality control were provided in Methods.
Replication	We replicated the ExWAS findings by examining another alcohol consumption GWAS that did not involve UK Biobank participants. Out of the 22 loci, 15 were successfully replicated at a nominal significance level.
Randomization	In the Exome-Wide Association Analysis, age, sex, and the first 10 genetic principal components (calculated with whole-exome sequencing data) were adjusted in the study. This study was an observational genetic epidemiology study without an interventional arm, and

randomization was therefore not relevant.

Blinding: Blinding was not applicable to this study as this study is observational.

Reporting for specific materials, systems and methods

We require information from authors about some types of materials, experimental systems and methods used in many studies. Here, indicate whether each material, system or method listed is relevant to your study. If you are not sure if a list item applies to your research, read the appropriate section before selecting a response.

Materials & experimental systems

n/a	Involved in the study
☒	☐ Antibodies
☒	☐ Eukaryotic cell lines
☒	☐ Palaeontology and archaeology
☒	☐ Animals and other organisms
☒	☐ Clinical data
☒	☐ Dual use research of concern
☒	☐ Plants

Methods

n/a	Involved in the study
☒	☐ ChIP-seq
☒	☐ Flow cytometry
☐	☒ MRI-based neuroimaging

Plants

Seed stocks: *Report on the source of all seed stocks or other plant material used. If applicable, state the seed stock centre and catalogue number. If plant specimens were collected from the field, describe the collection location, date and sampling procedures.*

Novel plant genotypes: *Describe the methods by which all novel plant genotypes were produced. This includes those generated by transgenic approaches, gene editing, chemical/radiation-based mutagenesis and hybridization. For transgenic lines, describe the transformation method, the number of independent lines analyzed and the generation upon which experiments were performed. For gene-edited lines, describe the editor used, the endogenous sequence targeted for editing, the targeting guide RNA sequence (if applicable) and how the editor was applied.*

Authentication: *Describe any authentication procedures for each seed stock used or novel genotype generated. Describe any experiments used to assess the effect of a mutation and, where applicable, how potential secondary effects (e.g. second site T-DNA insertions, mosiacism, off-target gene editing) were examined.*

Magnetic resonance imaging

Experimental design

Design type: Structural MRI

Design specifications: UK Biobank designed the imaging acquisition protocols including 6 modalities, covering structural, diffusion and functional imaging. The collection order is Tl-weighted structural image, resting-state functional MRI, task functional MRI, T2-weighted FLAIR structural image, Diffution MRI and susceptibility-weighted imaging. Tl-weighted structural image was acquired using straight sagittal orientation for 5 minutes.

Behavioral performance measures: Phenotypes data for alcohol intake were assessed from touchscreen questionnaires completed by the participants at the baseline assessment. The selected questions and corresponding Field ID for phenotypes used in this study were summarized in Supplementary Table 1.

Acquisition

Imaging type(s): T1-weighted structural imaging

Field strength: 3T

Sequence & imaging parameters: The EPI-based acquisitions utilize simultaneous multi-slice (multiband) acceleration. Biobank uses pulse sequences and reconstruction code from the Center for Magnetic Resonance Research (CMRR), University of Minnesota https:/ / www.cmrr.umn.edu/multiband. The resolution is lxlxl mm and field of view is 208x256x256 matrix. Straight sagittal orientation is used. TR and TE are 2000ms and 2.0lms respectively. The flip angle is 8 deg. Detailed sequence and imaging parameters are openly available here: https://biobank.ndph.ox.ac.uk/showcase/showcase/docs/ brain_mri.pdf

Area of acquisition: Whole brain

Diffusion MRI: ☒ Used ☐ Not used

Parameters	Diffusion-weighted images were acquired with two diffusion-weighted shells and 50 diffusion-encoding directions for each shell (i.e., with all 100 directions being distinct) at 2 × 2 × 2 mm (104 × 104 × 72 FOV matrix). b = 1000 shell (50 directions) was fed into the DTI fitting tool DTIFIT to estimate FA, tensor mode and MD.

Preprocessing

Preprocessing software	We used the pipeline with the Statistical Parametric Mapping software version 12 (http://www.fil.ion.ucl.ac.uk/spm) using the CAT12 toolbox (http://dbm.neuro.uni-jena.de/cat) with default settings, to preprocess the structural MRI data, which contained the usage of high-dimensional spatial normalization with an already integrated Dartel template in Montreal Neurological Institute (MNI) space. All images were subjected to nonlinear modulations and corrected for each individual head size. Images were then smoothed with an 8 mm full-width at half-maximum Gaussian kernel with the resulting voxel size of 1.5 mm3. The automated anatomical labeling 3 (AAL3) atlas, which partitioned the brain into 166 regions of interest, was employed to obtain the region-wise gray matter volume.
Normalization	See above
Normalization template	See above
Noise and artifact removal	See above
Volume censoring	See above

Statistical modeling & inference

Model type and settings	Mass univariate
Effect(s) tested	Associations of alcohol consumption-related genes and grey matter measures were tested using SKAT-O test through SAIGE-GENE+.

Specify type of analysis: ☒ Whole brain ☐ ROI-based ☐ Both

Statistic type for inference (See Eklund et al. 2016)	N/A (see methods on phenome-wide association analysis)
Correction	We applied Bonferroni correction for testing of multiple genes and traits

Models & analysis

n/a	Involved in the study
☒	☐ Functional and/or effective connectivity
☒	☐ Graph analysis
☒	☐ Multivariate modeling or predictive analysis
