# Supplementary material for: Whole exome sequencing analysis identifies genes for alcohol consumption
Source: Nat Commun. 2024 Jul 10;15:5777. doi: 10.1038/s41467-024-50132-3 (PMC11233704; doi:10.1038/s41467-024-50132-3)
Supplement: Supplementary file 6 — Source Data [file 41467_2024_50132_MOESM6_ESM.zip › SourceData/Source Data Description.docx]

Descriptions of Source Data used to plot figures in the manuscript of

Whole exome sequencing analysis identifies genes for alcohol consumption

**source data for Figure 2.xlsx:** Two tabs are provided. Figure2b is used to plot effect sizes and allele frequencies for significant variants, and Figure2c is used to plot percentages of various consequences of significant variants.

**source data for Figure 3.xlsx:** Four tabs are provided. Figure3a is used to plot the Manhattan plot showing the results of the rare variants (loss of function (LOF) and Missense) from ExWAS of alcohol consumption with three different MAF thresholds in gene-based analysis. Figure3b is used to plot the burden heritability of alcohol consumption in different groups from burden heritability regression analysis. Figure3c is used to plot the effect sizes of burden test of the significant associations. Figure3d is to plot the carrier percentage for rare LOF and missense variants in genes associated with alcohol consumption.

**source data for Figure 4.xlsx:** Two tabs are provided. Figure 4a is used to plot the results of the functional enrichment analysis. It contains the -log_10_ of p value (the negative_log10_of_adjusted_p_value column) of each GO term in the enrichment analysis. Figure 4b is used to plot the tissue-specific gene enrichment. It contains the fold-change values (the fold.change column) of the tissues.

**source data for Figure 5.xlsx:** Five tabs are provided. Figure5a is used to plot the top 10 genes with the most similar quantitative trait associations to *ANKRD12* derived from Gene-SCOUT. Figure5b is used to plot the enriched gene sets of *ANKRD12* plus the top 10 similar genes of *ANKRD12*. It contains the PHRED score (-10×log_10_(P)) (p_uncorrected column) for each gene set. Figure5c is used to plot gene expression of *ANKRD12* and *GIGYF1* in human tissues contained in the Human Protein Atlas database. It contains the normalised transcripts per million (nTPM column) for *ANKRD12* and *GIGYF1* in each tissue group. Figure5d is used to plot lifespan spatiotemporal expression trajectory of *ANKRD12* in the human brain. It contains log_2_ transformed expression value (y column) of *ANKRD12*. Figure5e is used to plot lifespan spatiotemporal expression trajectory of *GIGYF1* in the human brain. It contains log_2_ transformed expression value (y column) of *GIGYF1*.

**source data for Figure 6.xlsx:** Data to plot Figure 6. It contains the phenotypic associations of the rare-variant genes associated with alcohol consumption.
